# Osteoprotegerin deficiency aggravates methionine–choline-deficient diet-induced nonalcoholic steatohepatitis in mice

**DOI:** 10.1038/s41598-023-30001-7

**Published:** 2023-02-23

**Authors:** Shaobo Wu, Yao Wu, Lan Lin, Changshun Ruan, Fang Li, Rong Chen, Hongxin Du, Xianxiang Zhang, Xiaohe Luo

**Affiliations:** 1 Department of Laboratory Medicine, Chongqing University Three Gorges Hospital, School of Medicine, Chongqing University, No. 165, Xincheng Avenue, Wanzhou District, Chongqing, 404000 China; 2 The Center of Clinical Research of Endocrinology and Metabolic Diseases in Chongqing, Chongqing University Three Gorges Hospital, Chongqing, 404100 China; 3 Department of Endocrinology, Chongqing University Three Gorges Hospital, Chongqing, 404100 China; 4 Chongqing Municipality Clinical Research Center for Geriatric Diseases, Chongqing University Three Gorges Hospital, Chongqing, 404000 China

**Keywords:** Endocrine system and metabolic diseases, Gastrointestinal diseases

## Abstract

Clinical studies have shown that osteoprotegerin (OPG) is reduced in patients with nonalcoholic steatohepatitis (NASH), but the underlying mechanisms are unclear. The current study focuses on the role of OPG in the NASH pathogenesis. OPG knockout mice and wild-type control mice fed a methionine choline-deficient diet (MCD) for 4 weeks resulted in an animal model of NASH. Measurement of triglycerides (TG) in serum and liver to assess steatosis. Hematoxylin eosin (HE), Sirius Red and Masson staining were used to assess the liver damage. Transcriptome sequencing analysis, qPCR and western blot were to analyze changes in lipid metabolism and inflammation-related indicators in the liver. In vivo knockout of OPG resulted in a reduction of TG levels in the liver and a significant increase in serum ALT and AST. The expression of inflammatory factors and fibrosis genes was significantly upregulated in the livers of OPG knockout mice. Transcriptome sequencing analysis showed that OPG knockout significantly enhanced MCD diet-induced activation of the mitogen-activated protein kinase (MAPK) signaling pathway. Mechanistically, OPG may inhibit MAPK signaling pathway activity by upregulating the expression of dual specificity phosphatase 14 (DUSP14), thereby reducing inflammatory injury. OPG could regulate the activity of the MAPK signaling pathway via DUSP14, thus regulating the expression of some inflammatory factors in NASH, it may be a promising target for the treatment of NASH.

## Introduction

Nonalcoholic fatty liver disease (NAFLD) is a major global public health problem. Epidemiological studies have shown that this disease is prevalent in more than a quarter of people worldwide^1^. The spectrum of *NAFLD* ranges from simple steatosis (NAFL), steatohepatitis (NASH), and fibrosis, cirrhosis to liver cancer^2^. NASH is characterized by hepatocellular damage and inflammatory infiltration and is the key stage in NAFLD progression. Unfortunately, other than diet control and exercise, no appropriate treatment modalities are available, and only a few drugs have been approved for treating NASH^3^. Therefore, elucidating the molecular mechanism of NASH pathogenesis to develop NASH-related drugs is crucial.

Complex interorgan communication is involved in NAFLD pathogenesis, and various metabolic organs and tissues such as the liver, pancreas, adipose tissues, muscles, and bones are involved in its progression^4^. Previous studies have shown a possible interaction between NAFLD and bone metabolism^5^. Clinical data suggest that patients with NAFLD have a significantly lower bone mineral density (BMD) and are highly prone to fractures^6^. Conversely, as an endocrine organ, bones secret bone-derived factors that regulate local bone metabolism and systemic energy metabolic functions. Osteopontin (OPN), irisin, osteocalcin (OCN), and osteoprotegerin (OPG) probably mediate the interactions between bones, adipose tissues, and the liver^7^. Bone-derived factors including OPN and OCN play a role in NAFLD development^8^.

OPG is a member of the tumor necrosis factor (TNF) receptor superfamily^9^, which is present in various organs and tissues, including the liver, bone tissues, heart, and blood vessels. OPG is involved in various physiological and pathological processes, including bone metabolism, immune regulation, vascular function, and tumor metabolism. Clinical data indicate that OPG concentrations correlate with hypertension, left ventricular hypertrophy, vascular calcification, endothelial dysfunction, and the severity of liver injury in chronic hepatitis C^10^. OPG can function as soluble proteins because of the lack of transmembrane structures anchored to cell membranes. In osteoblasts, OPG regulates bone resorption via the OPG/receptor activator of the nuclear factor-κB (RANK)/RANK ligand (RANKL) pathway, thereby increasing BMD. Our previous study showed that OPG could regulate hepatic lipid degeneration via the extracellular signal-regulated kinases (ERK)/peroxisome proliferator-activated receptor gamma/CD36 pathway in the liver^11^. This suggests that OPG may be a key mediator of communication between the liver and bone tissues and between NAFLD and osteoporosis development. However, the role of OPG in NASH is not fully understood, and although some clinical studies have shown that OPG levels are significantly reduced in adults and children with NASH^12,13^, the underlying mechanism is unclear. Based on previous studies showing that OPG can regulate apoptosis and inflammation-related signals via several pathways including RANKL and TNF-related apoptosis-inducing ligand (TRAIL), we hypothesized that OPG could affect NASH progression by regulating inflammatory signals^14^. Therefore, in the present study, we analyzed the molecular mechanism of OPG in NASH by knocking out *TNFRSF11B *in vivo, combined with an animal model of NASH induced by a methionine- and choline-deficient (MCD) diet, to provide data for identifying promising targets for NASH treatment.

## Results

### OPG level is reduced in NASH

**Figure 1:**
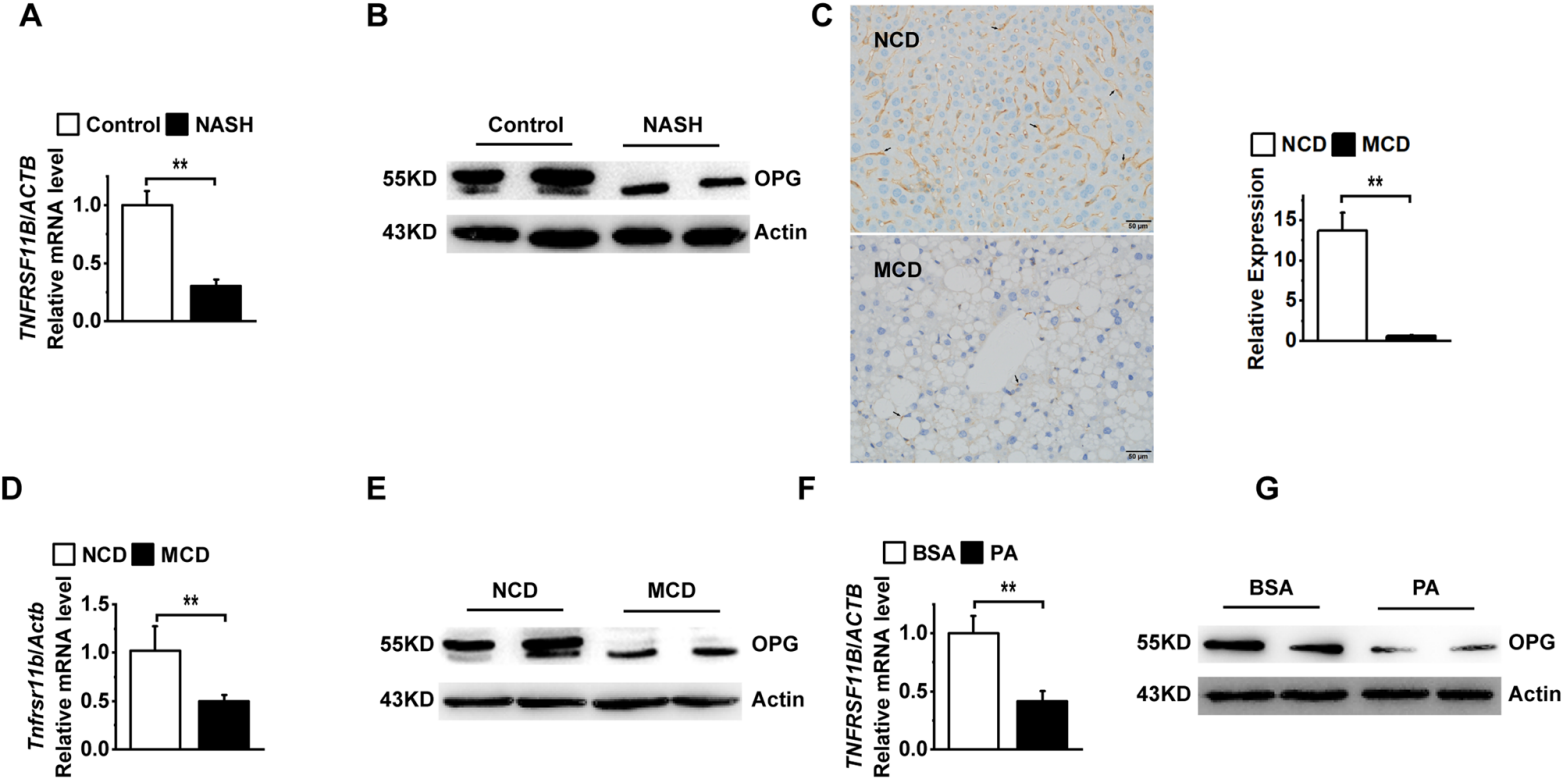
Osteoprotegerin (OPG) expression is downregulated in nonalcoholic steatohepatitis (NASH). (**A**) *TNFRSF11B* mRNA expression in the livers of patients with NASH. (**B**) OPG protein levels in the livers of patients with NASH. (**C**) Expression levels of OPG in liver tissues from methionine- and choline-deficient (MCD) diet-induced NASH animal model. Scale bars: 50 μm. OPG are shown by arrows. Graph showing comparisons of OPG expression in liver tissues from control or MCD diet-induced mice. (**D**) *Tnfrsf11b* mRNA expression in the livers of the methionine- and choline-deficient (MCD) diet-induced NASH animal model. (**E**) OPG protein levels in the livers of the MCD-induced NASH animal model. (**F**) *TNFRSF11B* mRNA expression in the PA-induced NASH cell model. (**G**) OPG protein levels in the PA-induced NASH cell model. Each group at least has 3 samples. The data in (**A**), (**C**), (**D**), and (**F**) are presented as the mean ± SD, **p < 0.01.

We analyzed *TNFRSF11B* expression in the liver of patients with NASH to investigate the role of OPG and found that the *TNFRSF11B* mRNA level was reduced in patients with NASH (Fig. 1A). The western blotting and immunohistochemical results showed that the OPG level was also significantly reduced in patients with NASH (Fig. 1B,C). In the animal model of NASH induced by the MCD diet, *Tnfsrf11b* mRNA expression and OPG levels were also significantly reduced (Fig. 1D,E). In PA-treated hepatocytes, *TNFRSF11B* mRNA and OPG levels were also significantly reduced (Fig. 1F,G). These results suggested that OPG may play a critical role in NASH development.

### OPG deficiency reduced MCD diet-induced hepatic steatosis

**Figure 2:**
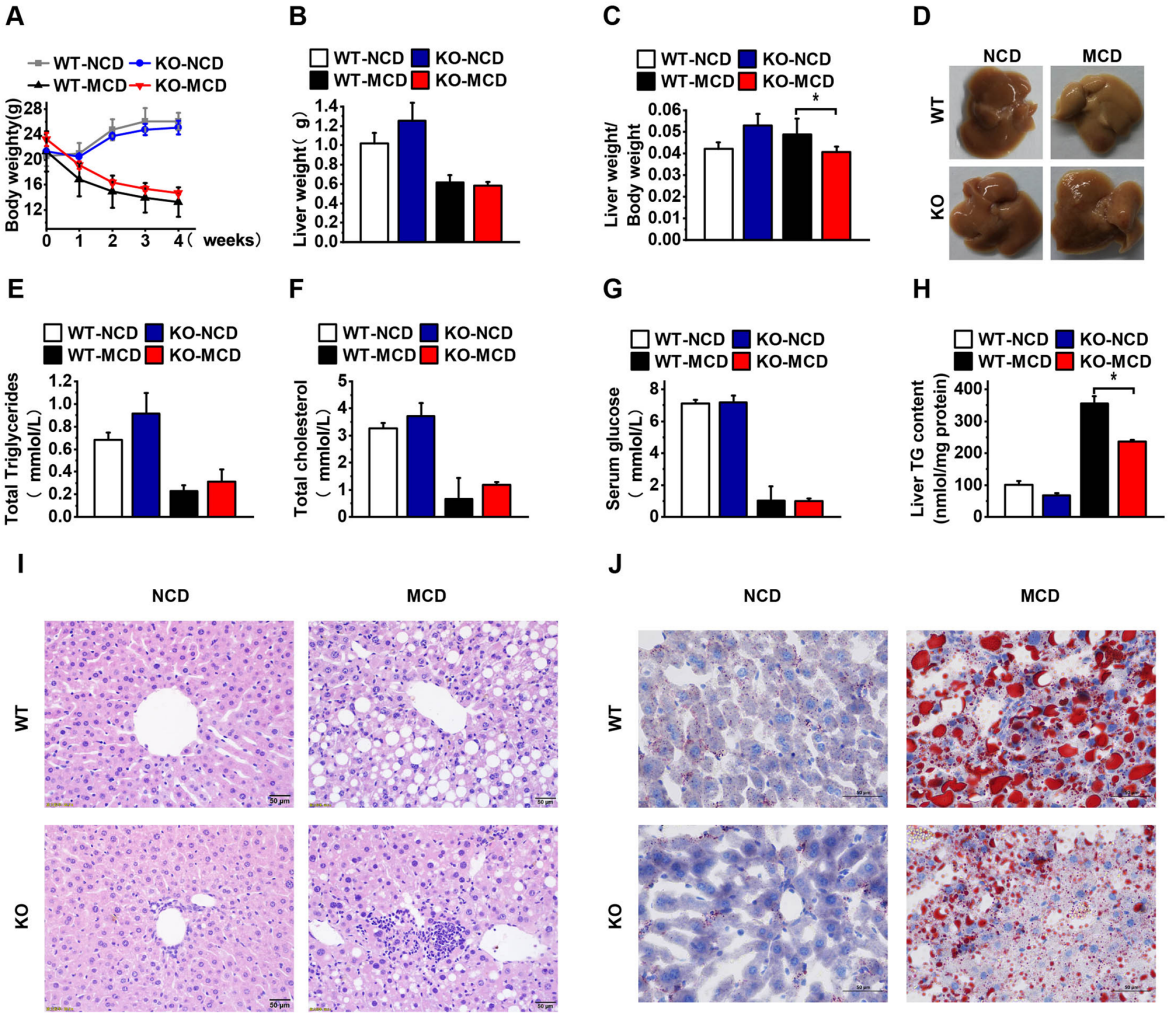
Osteoprotegerin (OPG) deficiency reduced methionine- and choline-deficient (MCD) diet-induced hepatic steatosis. (**A**) The body weight of the WT or OPG KO mice after MCD diet 1–4 weeks. (**B**) Liver weight of the mice. (**C**) Liver weight to body weight ratio. (**D**) Gross photograph of the mice’s liver. (**E**) Serum triglyceride levels. (**F**) Total serum cholesterol. (**G**) Serum glucose. (**H**) Triglyceride levels in the liver. (**I**) The representative image of hematoxylin–eosin staining. (**J**) The representative image of Oil Red O staining. The scale bar is 50 µm. Each group at least has 3 samples. The data in (**A**–**C**), (**E**–**H**) are presented as the mean ± SD, *p < 0.05.

**Figure 3:**
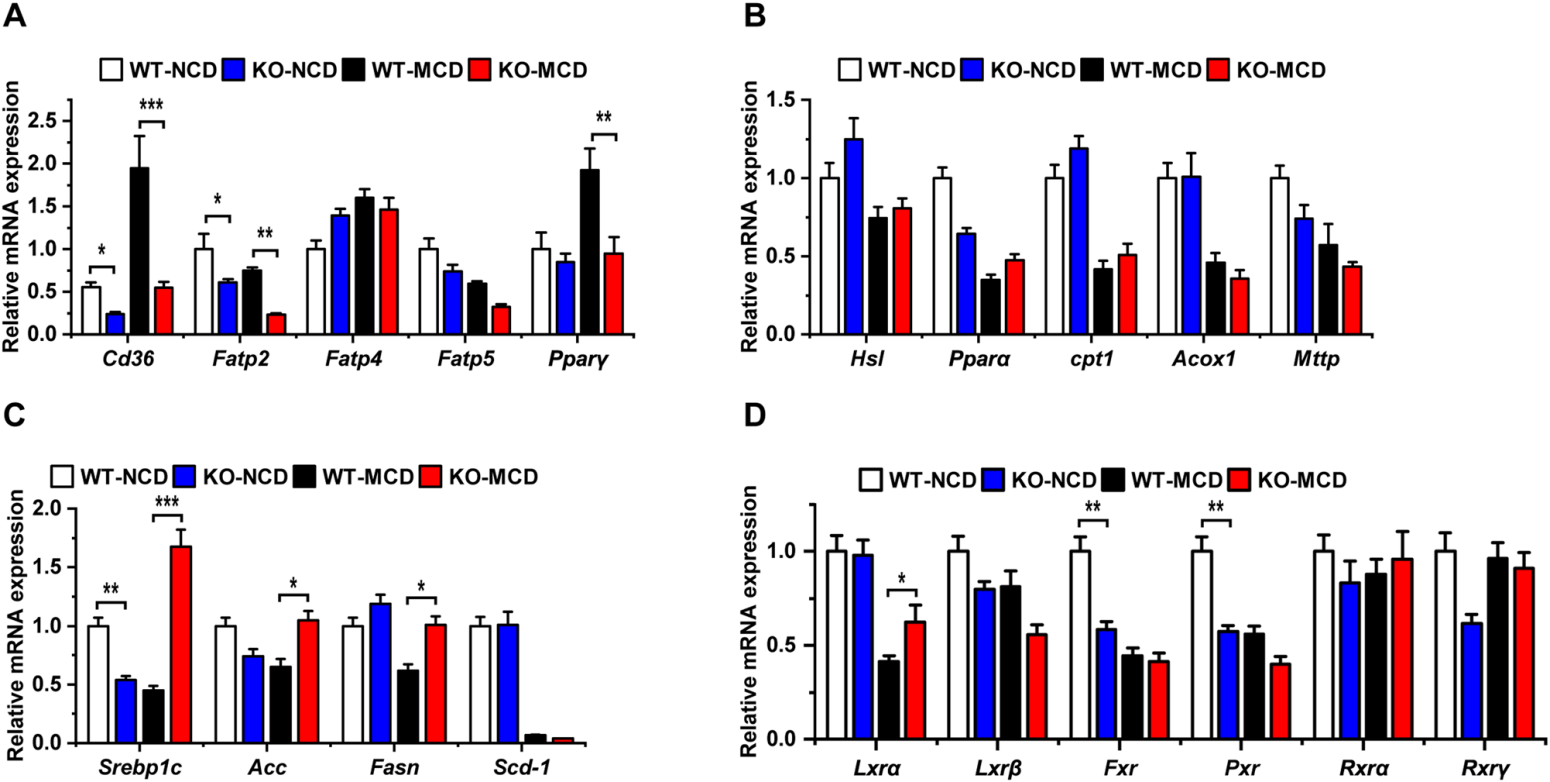
Lipid metabolism gene expression in liver tissues. (**A**) Fatty acid uptake gene expression. (**B**) Fatty acid oxidation gene expression. (**C**) Fatty acid synthesis gene expression. (**D**) Nuclear receptor gene expression. Each group at least has 3 samples. The data in (**A**–**D**) are presented as the mean ± SD, *p < 0.05; **p < 0.01, and ***p < 0.001.

We constructed OPG-knockout (KO) mice to study the function of OPG in vivo. After 4 weeks of feeding on the MCD diet, the mice showed a significant decrease in body and liver weights (Fig. 2A,B, Supplement Fig. 1). The ratio of liver weight to body weight was reduced in the KO group compared with that in the wild-type (WT) control group consuming the MCD diet (Fig. 2C). Gross liver photographs showed significant liver damage after MCD feeding, and the damage was more pronounced in the KO group (Fig. 2D). Serum TG, TC, and blood GLU concentrations were significantly lower in the MCD-fed group; however, no significant difference in these parameters was observed between the KO and WT control groups (Fig. 2E–G). The liver TG levels were significantly increased after MCD feeding but were lower in the KO group than in the WT control group (Fig. 2H). HE staining and Oil Red O staining showed less vacuolar degeneration and lipid droplet aggregation in the livers of the KO group of mice (Fig. 2I,J). These results suggested that OPG knockdown reduced hepatic steatosis induced by the MCD diet. To further analyze the cause of liver steatosis, we extracted liver tissue mRNA and performed a quantitative PCR analysis, which showed that the expressions of fatty acid uptake genes *Cd36* and *Fatp2,* as well as that of *Pparγ* were significantly downregulated in the KO group, whereas the expression of *Fatp4* and *Fatp5* remained unchanged (Fig. 3A). The expressions of the fatty acid synthesis genes *Srebp1c*, *Acc1*, and *Fasn* were upregulated in the KO mice (Fig. 3C). The expression of the oxidation genes *Pparα*, *Cpt1*, *Acox1*, and *Mttp* remained unchanged (Fig. 3B), and the expression of *Lxrα* were significantly upregulated in the KO group, whereas the expression of the other nuclear receptor genes *Lxrβ*, *Fxr*, *Pxr*, *Rxrα*, and *Rxrγ* remained unchanged (Fig. 3D). These results suggested that OPG affects the hepatic TG levels primarily by repressing fatty acid synthesis and facilitating fatty acid intake.

### OPG deficiency exacerbated MCD diet-induced liver injury

**Figure 4:**
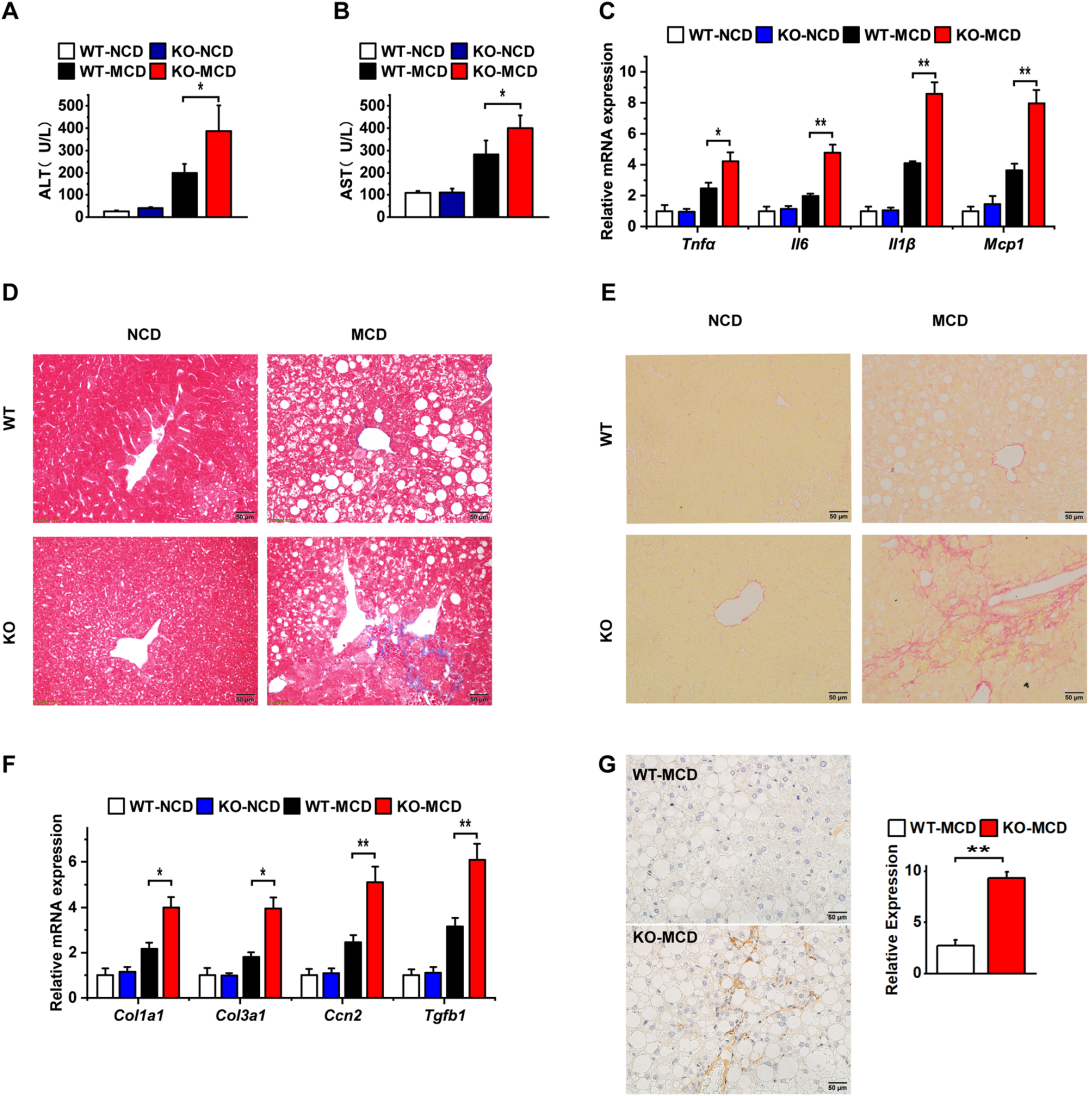
Osteoprotegerin (OPG) deficiency exacerbated methionine- and choline-deficient (MCD) diet-induced liver injury. (**A**) Serum alanine aminotransferase levels. (**B**) Serum aspartate aminotransferase levels. (**C**) Inflammatory gene expression. (**D**) The representative image of Masson staining. (**E**) The representative image of Sirius red dye. (**F**) Fibrosis gene expression. (**G**) αSMA immunohistochemical assay. The scale bar is 50 µm. Graph showing comparisons of αSMA expression in liver tissues from MCD diet-induced WT or OPG KO mice. Each group at least has 3 samples. The data in (**A**–**C**), (**F**,**G**) are presented as the mean ± SD, *p < 0.05; **p < 0.01.

NASH is characterized by the occurrence of inflammatory infiltration and hepatocellular damage compared with simple steatosis^3^. HE staining of the tissue sections revealed that significant inflammatory cell infiltration occurred in the liver tissues after the mice were fed the MCD diet, and more inflammatory cell aggregation occurred in the OPG KO group than in the WT control group (Fig. 2I). Thus, we measured the serum levels of ALT and AST to assess the extent of hepatocyte damage. In the KO group of mice, ALT and AST levels were more significantly increased (Fig. 4A,B). The infiltration of inflammatory cells is often accompanied by changes in the expression of inflammatory factors; hence, we examined the expression of conventional inflammatory factors, including TNFα (*Tnfα*), interleukin (IL) 6 (*Il6*), IL1β (*Il1β*), and monocyte chemoattractant protein-1 (*Mcp-1*). After the mice were fed the MCD diet, the mRNA expression of the inflammatory factors was significantly upregulated and was higher in the KO group than in the WT control group (Fig. 4C). The development of liver fibrosis is a key pathological feature of NASH progression^3^. Masson staining and Sirius red staining showed that fibrosis was more severe in the KO group of mice than in the WT control group (Fig. 4D,E). Collagen fiber-related gene expression was significantly upregulated in the KO mice (Fig. 4F). Immunohistochemical results showed that the amount of α smooth muscle actin (SMA) was significantly increased in the KO group (Fig. 4G). These results suggested that OPG KO exacerbated liver damage.

### OPG deficiency exacerbated MCD diet-induced liver injury via mitogen-activated protein kinase (MAPK) signaling

**Figure 5:**
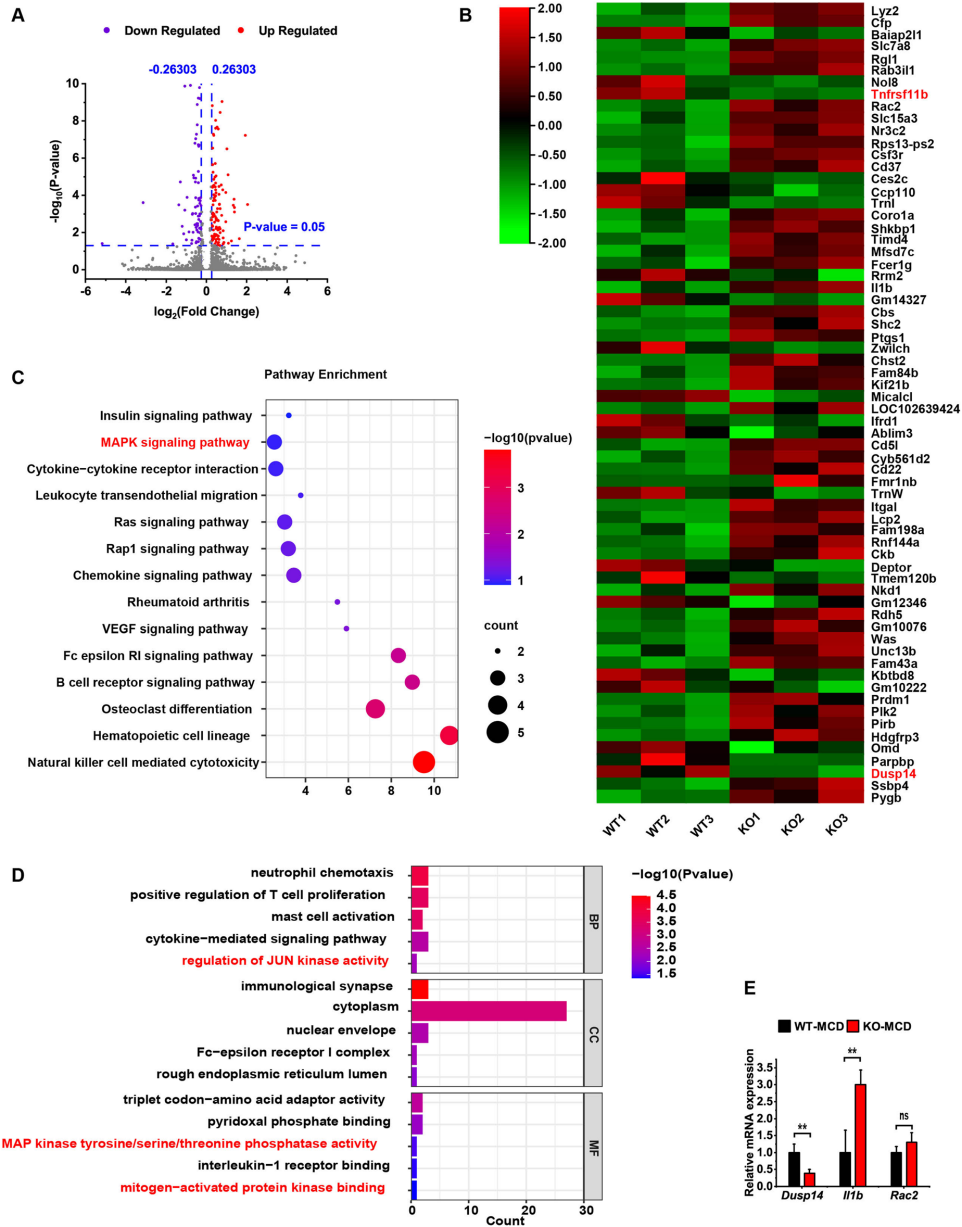
Analysis of the liver transcriptome by second-generation sequencing. (**A**) Volcano map of differentially expressed genes. (**B**) Heatmap of differentially expressed genes. (**C**) Bubble diagram of Kyoto Encyclopedia of Genes and Genomes (KEGG) enrichment analysis. (**D**) Bar chart of gene ontology analysis. (**E**) Quantitative polymerase chain reaction results. Each group at least has 3 samples. The map was plotted using TBtools software^15^ (version 1.0986; https://github.com/CJ-Chen/TBtools/releases) and a free online platform for data visualization (http://www.bioinformatics.com). Differential gene expression and statistical significance were analyzed using DESeq2 package (http://www.bioconductor.org/). The data in (**E**) are presented as the mean ± SD. ns, no significant difference; **p < 0.01.

We performed transcriptome sequencing analysis to explore the molecular mechanisms through which OPG regulates NASH. The principal component analysis revealed that the expression matrices of the KO and WT groups had two distinct dimensions (Supplement Fig. 2). The differential gene expression analysis revealed 160 genes with remarkable changes (Fig. 5A), including 66 genes with large fold changes (Fig. 5B). The Kyoto Encyclopedia of Genes and Genomes (KEGG) functional enrichment analysis revealed that the MAPK signaling pathway was altered in the KO group (Fig. 5C). Gene ontology (GO) analysis also showed that OPG could regulate the MAPK kinase binding capacity and its tyrosine, serine, and threonine phosphatase activities (Fig. 5D). These results suggested that OPG can regulate the MAPK signaling pathway.

**Figure 6:**
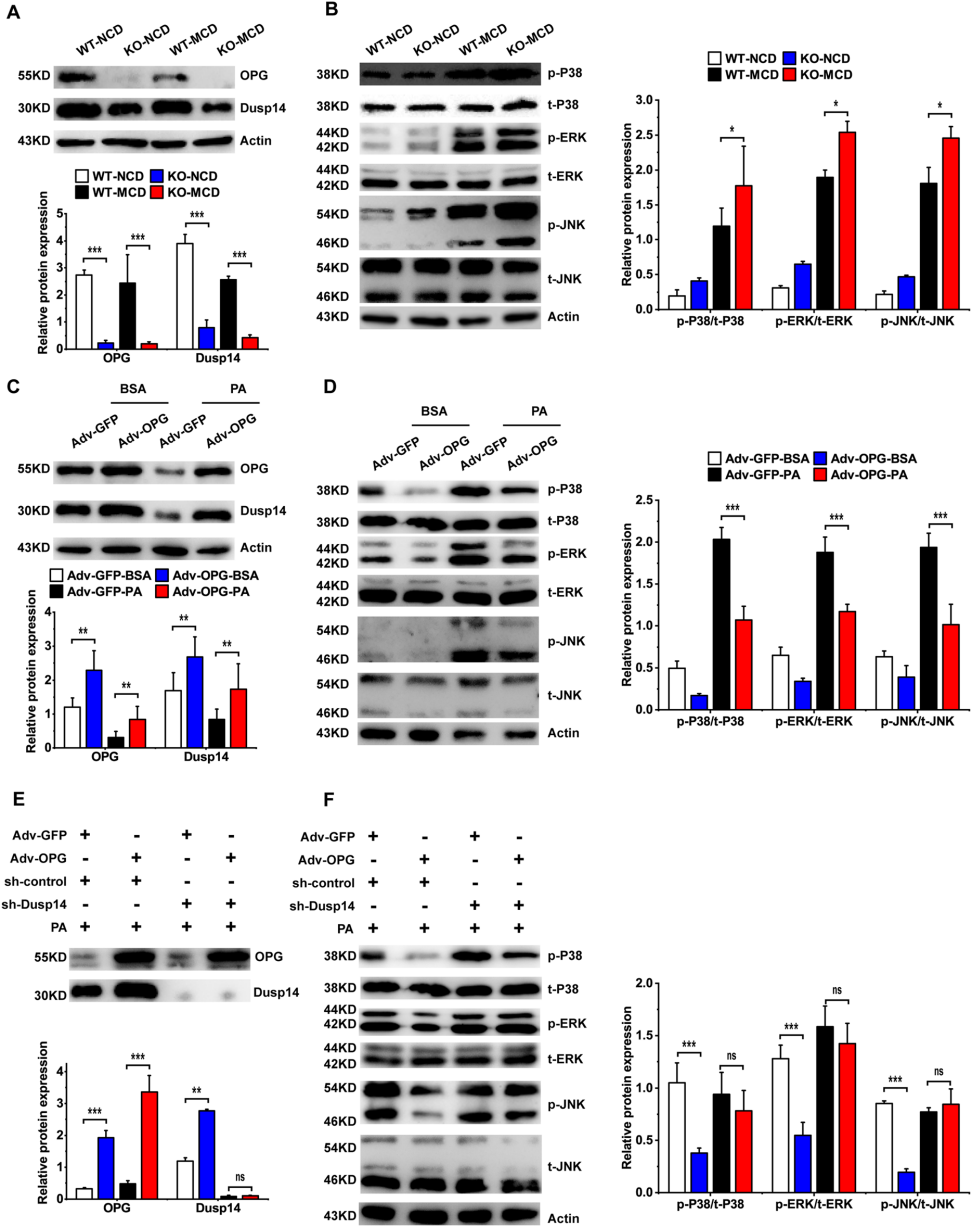
Osteoprotegerin (OPG) regulates MAPK signaling pathway activity via DUSP14. (**A**) OPG and dual specificity phosphatase 14 (DUSP14) protein levels in the mice. (**B**) Phosphorylation levels of extracellular signal-regulated kinases (ERK), c-Jun N-terminal kinase 1 (JNK), and P38 in mouse liver. (**C**) OPG and DUSP14 proteins in OPG-overexpressing hepatocytes. (**D**) Phosphorylation levels of ERK, JNK, and P38 in OPG-overexpressing hepatocytes. (**E**) OPG and DUSP14 proteins in DUSP14 knockout hepatocytes. (**F**) Phosphorylation levels of ERK, JNK, and P38 in DUSP14 knockout hepatocytes. Each group at least has 3 samples. The data in (**A**–**F**) are presented as the mean ± SD; *ns* no significant difference, *p < 0.0, **p < 0.01, and ***p < 0.001.

DUSP14 is a ubiquitously present phosphatase that dephosphorylates ERK, c-Jun N-terminal kinase 1 (JNK), and p38 in the MAPK signaling pathway to regulate the inflammatory response in the liver. In the transcriptome sequencing results, *Dusp14* expression was significantly downregulated in the KO group (Fig. 5B). qRT-PCR confirmed that *Dusp14* expression changed significantly in the KO group (Fig. 5E). The western blot analysis showed that OPG was largely undetectable in the KO group, and consistent with the mRNA expression, DUSP14 content was significantly reduced after the KO of OPG (Fig. 6A). Hepatic ERK, JNK, and P38 activities were significantly enhanced after the mice were fed the MCD diet, and the activity in the KO group was significantly higher than that in the WT control group (Fig. 6B). To confirm that OPG can regulate *DUSP14* expression and affect MAPK signaling, we overexpressed *TNFRSF11B* in L02 cells in an adenovirus-mediated manner. In hepatocytes, OPG overexpression significantly increased DUSP14 content and decreased MAPK signaling pathway activity (Fig. 6C,D). Based on the aforementioned experimental results, we speculated that OPG affected the MAPK signaling pathway by regulating *DUSP14* expression. We tested this hypothesis by constructing an interfering RNA targeting *DUSP14* and experimentally found that OPG could not reduce the activity of the MAPK signaling pathway after pre-inhibiting *DUSP14* expression (Fig. 6E,F). These results suggested that OPG can affect the activity of the MAPK signaling pathway by regulating *DUSP14* expression.

## Discussion

In the present study, we found that the hepatocyte OPG level was significantly decreased in patients with NASH, NASH animal models, and cellular models. Most clinical studies have shown decreased circulating-OPG levels in patients with NASH^12,16,17^; however, one study has reported increased OPG levels^18^. These differences may be attributed to factors including ethnicity, different diagnostic criteria, and different manufacturers of test kits. Differences in OPG levels in NASH suggest its role in the disease. Whether a decrease in OPG levels promotes the development of NASH or a progressive process of NASH leads to a decrease in OPG levels remains to be investigated. To better explore the role of OPG in NASH, we used the MCD diet-induced NASH animal model. However, this animal model has some limitations such as the absence of clinical patient manifestations of obesity and insulin resistance, as well as marked weight loss^19^. However, the MCD animal model has several key pathological features of NASH such as steatosis and inflammation^20^. After 1 day of consuming the MCD diet, the mice began to lose body weight significantly. After 4 weeks of consuming the MCD diet, liver lipid aggregation was evident, and the liver fat content of the OPG KO mice was lower than that of the WT controls. These results are consistent with our previous results obtained after feeding the mice with 45% high-fat diet (HFD)^11^. Other key features of MCD-diet consumption are the increased lipid degradation in adipose tissues and increased lipid uptake in liver tissues^21^. The downregulation of CD36 expression resulted in a relatively low TG content in the liver of the OPG KO mice.

The most important pathological feature of NASH relative to simple steatosis is the occurrence of liver injury and inflammatory infiltration^22,23^. Sodium glucose co-transporter2 inhibitors could attenuate the inflammation process^24,25^. The present results showed more severe hepatocellular injury in the OPG KO mice after they were fed the MCD diet. The expressions of the inflammatory factors *Tnfα*, *Il6*, and *Il1β* were significantly upregulated, which could partially explain the liver injury induced by OPG knockdown. OPG, a member of the TNF receptor superfamily, plays a vital role in regulating inflammatory factors. Unlike traditional TNF family members, OPG functions as a secretory receptor because it lacks the transmembrane structure. Circulating OPGs are altered in various inflammatory diseases^26,27^. Interestingly, inflammatory factors such as IL1β can also stimulate OPG secretion^28^. These studies suggest that a complex modality of regulation exists between OPG and inflammatory factors. Previous studies have shown that OPG can regulate inflammation-related pathways via pathways including OPG/RANK/RANKL and TRAIL^29–31^. However, the transcriptional sequencing analysis showed that OPG KO affected the activity of the MAPK signaling pathway. The MAPK signaling pathway plays a major role in inflammation in various experimental models of NASH, and sustained activation of the MAPK signaling pathway in patients with NASH has been observed^32,33^. Three well-characterized MAPK subfamilies are p38, JNK, and ERK1/2, which are related to NASH development. MAPK can be activated by upstream kinase signals, such as apoptosis signal-regulated kinase 1 and mixed-spectrum kinase 3^34,35^. By contrast, MAPKs are inactivated through direct dephosphorylation of their threonine and tyrosine residues by a set of bispecific protein tyrosines^36–38^. DUSPs, also called MAPK phosphatases, play a key role in regulating MAPK activity^39^. DUSP14 is a ubiquitously present phosphatase containing a highly conserved C-terminal catalytic domain that confers phosphatase activity^40^. DUSP14 negatively regulates the activity of ERK, JNK, and p38^41–43^. DUSP14 significantly ameliorates HFD-mediated or genetically induced insulin resistance, hepatic steatosis, and concomitant inflammation. Furthermore, the regulation of hepatic energy homeostasis by DUSP14 is mediated by direct interaction with transforming growth factor (TGF) β-activated kinase 1 (TAK1) and the subsequent inhibition of TAK1 and its downstream signaling pathways^44^. In the present study, OPG KO significantly reduced the DUSP14 levels. OPG could regulate the activity of the MAPK signaling pathway via DUSP14, as verified by in vitro experiments.

Interestingly, the expressions of the fibrosis markers Col1a1, Col3a1, Ccn, Tgfβ, and αSMA were also upregulated in the OPG KO mice. However, clinical studies have shown increased OPG levels in fibrotic organs, including the liver, lung, heart, blood vessels, and kidney^45^. The underlying molecular mechanism may be that OPG binds to integrins to increase the release of TGFβ. In the present study, we used short-term MCD-fed mouse model and the probability of hepatic fibrosis in the liver was low and did not mimic clinical liver fibrosis well. Moreover, the liver develops more severe hepatic fibrosis by activating mesenchymal cells such as hepatic stellate cells. We did not isolate hepatocytes, macrophages, or hepatic stellate cells to study them separately.

This study has some limitations. First, we used OPG-whole-body-KO mice and did not use liver-conditional-KO mice for the reasons we have described in our previous study^46^. Second, we used only the MCD diet-induced model, which has some limitations as previously described. In subsequent studies, various NASH animal models can be used for validation, including high-fat and -cholesterol diet, HFD, and western diet models, carbon-tetrachloride-induced fibrosis models, and total bile duct ligation models. Previous reports have shown the role of OPG in fibrosis, and we intend to follow up with a special fibrosis induction model to study the effect of OPG on fibrosis in NASH. Finally, we only verified that OPG could regulate the MAPK pathway activity via DUSP14; however, we could not explore in detail the specific molecular mechanism by which OPG affects DUSP14 expression.

## Conclusions

In the present study, we found that OPG could regulate the activity of the MAPK signaling pathway via DUSP14, thus regulating the expression of some inflammatory factors in NASH, indicating that it may be a promising target for the treatment of NASH. We aim to subsequently elucidate the molecular mechanism of OPG-mediated DUSP14 regulation in depth to explore the NASH development and treatment.

## Materials and methods

### Animal studies

OPG knockout mice (C57BL/6J, OPG^−/−^) were obtained from Southern Model Animal Center (Shanghai, China)^11^. Eight-week-old male mice were acclimatized to the environment for 1 week before initiating the experiments. Male OPG^+/+^ and OPG^−/−^ mice (9 weeks old) were fed with either a normal control diet (NCD, #MD12051, Medicine Inc. Jiangsu, China) or a methionine–choline-deficient diet (MCD, #MD12052, Medicine Inc. Jiangsu, China) for 4 weeks. The mice were maintained in a 12 h/12 h light/dark cycle. They were weighed at the same time each week. After feeding for 4 weeks, the mice were starved for 6–8 h, and then collected mice blood from the retro-orbital plexus. Isolated liver tissues were snap-frozen in liquid nitrogen. All animal protocols were approved by the Animal Care and Use Review Committee of Chongqing University Three Gorges Hospital (Chongqing, China).

### Human liver tissue

Liver specimens of NASH and non-NASH patients who underwent biopsy were collected from the Department of Pathology, Chongqing University Three Gorges Hospital. All operations were performed in accordance with the Helsinki Declaration protocol and were approved by the Ethics Committee of Chongqing University Three Gorges Hospital (Chongqing, China).

### Cell culture

Hepatocytes were cultured in Dulbecco’s Modified Eagle Medium (DMEM, Gibico™, Cat.No.10567022) containing 10% fetal bovine serum (FBS, Gibico™, Cat.No. 10100147). For palmitic acid (PA, KunChuang Co. Ltd., Xi’an, China) treatment, approximately 8.5 × 10^5^ L02 cells were spread flat in each well of a 6-well plate and cultured overnight in a humidified incubator with 5% CO_2_ at 37 °C. Cells at 80–90% confluence were treated with 0.75 mM PA for 24 h. For plasmid transfection, approximately 7.5 × 10^5^ L02 cells were placed into each well of a 6-well plate and incubated overnight at 37 °C in the presence of 5% CO_2_. In 60–70% confluent cells, dual specificity phosphatase 14 (*DUSP14*)-short hairpin RNA (shRNA) or control shRNA was transfected using Lipofectamine 3000 reagent (Invitrogen™ Cat. No. 3000015). For viral infection, approximately 7.5 × 10^5^ L02 cells were spread flat in each well of a 6-well plate and incubated overnight at 37 °C in the presence of 5% CO_2_. As described previously^11^, adenovirus-overexpressing OPG was added at MOI = 50, and polybrene was added at a final concentration of 5 µg/mL for 24 h.

### Serum biochemical measurements

For serum biochemistry testing, mouse sera were obtained and analyzed at the Department of Laboratory Medicine, Chongqing University Three Gorges Hospital, for various indicators, including alanine aminotransferase (ALT), aspartate aminotransferase (AST), triglyceride (TG), total cholesterol (TC), and glucose (GLU).

### Liver TG content

The TG content in the liver was determined using an optimized GPO Trinder enzymatic reaction (Cat. No. E1025-105, Applygen Technologies Inc., Beijing, China). The procedure was performed according to the manufacturer’s instructions. Briefly, 50 mg of liver tissue was weighed, and 1 mL of lysate was homogenized. An appropriate amount of supernatant was collected for protein quantification using a BCA kit (Cat. No. P0012S, Beyotime Biotechnology, China). Another sample of supernatant was heated at 70 °C for 10 min and then centrifuged at 2000 rpm for 5 min, and the supernatant was collected for enzymatic assays.

### Hematoxylin and eosin staining

Fresh liver tissue was prepared into paraffin blocks by fixation, dehydration, and embedding. Paraffin sections of 5-μm thickness were dewaxed and used for hematoxylin and eosin (HE) staining, which was performed according to the manufacturer’s instructions of a commercial kit (#G1120, Beijing Solarbio Science and Technology Co., Ltd., China).

### Oil Red O staining

Fresh liver tissue was prepared into frozen sections of 5-μm thickness. The sections were fixed using 4% paraformaldehyde for 5 min, washed with isopropyl alcohol, and stained with 60% Oil Red O stain for 10–15 min, followed by hematoxylin restaining and sealing of the sections for photographs. The staining kit was purchased from Beijing Solarbio Science and Technology (#G1261, China).

### Masson’s trichrome staining

Fresh liver tissue was prepared into paraffin blocks by fixation, dehydration, and embedding. Paraffin sections of 5-μm thickness were dewaxed and used for Masson staining, which was performed according to the manufacturer’s instructions of a commercial kit (#G1340, Beijing Solarbio Science and Technology Co., Ltd., China).

### Sirius Red staining

Liver tissue sections (6-μm-thick) were dewaxed and stained with Sirius Red stain for 1 h, restained with hematoxylin, dehydrated, cleared, and sealed with neutral gum. The staining kit was purchased from Beijing Solarbio Science and Technology (#G3632, China).

### Immunohistochemistry

Paraffin sections were dewaxed and covered with water, and antigen repair was then performed using sodium citrate. Next, peroxidase activity in the tissues was inhibited with 3% H_2_O_2_, and nonspecific binding was blocked with 5% normal goat serum. Immunoreactivity was detected using a monoclonal antibody against mouse anti-OPG (#sc-390518, Santa Cruz Biotechnology), followed by secondary antibody color development, and finally, hematoxylin restaining and neutral resin blocking. The staining kit was purchased from Shenzhen NeoBioscience and Technology (ENS003.300, China).

### Quantitative polymerase chain reaction

Total RNA was extracted using TRIzol reagent (#9109, Takara, Japan), and reverse transcription was performed using a reverse transcription kit (#G3337, Seville, China). Real-time quantitative polymerase chain reaction (qRT-PCR) was performed using a real-time PCR system (Jena, qTOWER2, Germany). The primers used for qRT-PCR are presented in Supplementary Table 1.

### Western blot analysis

The western blot procedure is described in detail in our previous study^11^. The blots were cut prior to hybridization with primary antibodies during blotting. Primary antibodies included anti-OPG (#sc-390518, Santa Cruz Biotechnology), anti-DUSP14 (#ab272587, Abcam), anti-extracellular signal-regulated kinase (ERK, # 4695, Cell Signaling Technology), anti-phospho-ERK (#4370, Cell Signaling Technology), anti-P38 (#9212, Cell Signaling Technology), anti-phospho-P38 (#4511, Cell Signaling Technology), anti-c-Jun NH2-terminal kinase (JNK, #9252, Cell Signaling Technology), anti-phospho-JNK (#4668, Cell Signaling Technology), and anti-β-actin (#TA-09, ZSGB-BIO, China) antibodies. Secondary antibodies included Goat Anti-Rabbit IgG(H + L) HRP (GAR007, MULTI SCIENCES, China) and Goat Anti-Mouse IgG(H + L) HRP (GAM007, MULTI SCIENCES, China).

### RNA-sequencing

RNA-Sequencing experiments were performed by an experimental expert in the laboratory of Novel Bio Co., Ltd. The tissues of the model mice were surgically removed and stored at − 80 °C. The data were analyzed by NovelBio Co., Ltd. using the NovelBrain Cloud Analysis Platform (http://www.novelbrain.com). Heatmap was plotted using TBtools software^15^ (version 1.0986; https://github.com/CJ-Chen/TBtools/releases) and a free online platform for data visualization (http://www.bioinformatics.com). Differential gene expression and statistical significance were analyzed using DESeq2 package (http://www.bioconductor.org/).

### Statistical analysis

Data are presented as the mean ± standard deviation (SD) or standard error (SE). Statistical analyses were performed using SPSS (version 23) and Prism (Free Trial Version 8.0.1) software. Statistical significance was assessed through Student’s *t* test, and the significance of differences among more than two groups was determined by one-way analysis of variance. A p value < 0.05 was considered statistically significant.

### Ethics approval

The study was conducted in accordance with the Helsinki Declaration and all animal protocols were approved by the Animal Care Committee of Chongqing University Three Gorges Hospital (Chongqing, China), and also followed the Guide for the Care and Use of Laboratory Animals and ARRIVE guidelines.

## Supplementary Information

Supplementary Information.

## Data Availability

All data generated or analyzed during this study are available from the corresponding author on reasonable request.

## Abbreviations

OPG: Osteoprotegerin
NASH: Nonalcoholic steatohepatitis
qPCR: Quantitative PCR
MCD: Methionine–choline-deficient diet
ALT: Alanine aminotransferase
AST: Aspartate aminotransferase
HE: Hematoxylin eosin
MAPK: Mitogen-activated protein kinase
DUSP14: Dual specificity phosphatase 14
NAFLD: Nonalcoholic fatty liver disease
NAFL: Simple steatosis
BMD: Bone mineral density
OPN: Osteopontin
OCN: Osteocalcin
RANK: Receptor activator of nuclear factor-kB
RANKL: Receptor activator of nuclear factor-kB ligand
ERK: Extracellular signal-regulated kinase
PPARγ: Peroxisome proliferator-activated receptor gamma
CD36: Cluster of differentiation 36
NCD: Normal control diet
OPG^−^^/^^−^: OPG knockout mice
DMEM: Dulbecco's Modified Eagle Medium
PA: Palmitic acid
shRNA: Short hairpin RNA
MOI: Multiplicity of infection
TG: Triglyceride
TC: Total cholesterol
GLU: Glucose
GPO: L-α-Glycerophosphate oxidase
BCA: Bicinchoninic acid assay
JNK: C-Jun NH2-terminal kinase
SD: Standard deviation
SE: Standard error
ANOVA: One-way analysis of variance
Fatp2: Fatty acid transporter protein2
Fatp5: Fatty acid transporter protein5
Srebp1c: Sterol regulatory element binding proteins 1c
Acc1: Acetyl CoA carboxylase
Fasn: Fatty acid synthase
Pparα: Peroxisome proliferator-activated receptor alpha
Scd1: Stearoyl CoA desaturase 1
Cpt1: Carnitine palmitoyltransferase 1
Acox1: Acyl-CoA oxidase 1
Mttp: Microsomal triglyceride transfer protein
LXRα: Liver X receptor alpha
LXRβ: Liver X receptor beta
FXR: Farnesoid X receptor
PXR: Pregnane X receptor
RXRα: Retinoid X receptor
RXR: Retinoid X receptor
Tnfα: Tumor necrosis factor α
Il6: Interleukin 6
Il1β: Interleukin 1β
Mcp-1: Monocyte chemoattractant protein-1
αSMA: Alpha-smooth muscle actin
TRAIL: Tumor necrosis factor-related apoptosis-inducing ligand
ASK1: Apoptosis signal-regulated kinase 1
MLK3: Mixed-spectrum kinase 3
MKPs: MAPK phosphatases
TAK1: Transforming growth factor β activated kinase 1
Col1a1: Collagen type I alpha 1 chain
Col3a1: Collagen type III alpha 1 chain
Ccn: Cellular communication network 2
Tgfβ: Transforming growth factor beta
KEGG: Kyoto encyclopedia of genes and genomes
