# Supplementary material for: Osteoprotegerin deficiency aggravates methionine–choline-deficient diet-induced nonalcoholic steatohepatitis in mice
Source: Sci Rep. 2023 Feb 23;13:3194. doi: 10.1038/s41598-023-30001-7 (PMC9950492; doi:10.1038/s41598-023-30001-7)
Supplement: Supplementary file 1 — Supplementary Information. [file 41598_2023_30001_MOESM1_ESM.pdf]

Osteoprotegerin deficiency aggravates methionine-choline-deficient diet-induced nonalcoholic steatohepatitis in mice

Shaobo Wu[2,3#], Yao Wu[1#], Lan Lin[1], Changshun Ruan[2,3], Fang Li[1], Rong Chen[1], Hongxin Du[1], Xianxiang Zhang[2,3 *], Xiaohe Luo[1,2,4 *]

[1]Department of Laboratory Medicine, Chongqing University Three Gorges Hospital, School of Medicine, Chongqing University, Chongqing 404000, China

[2]The Center of Clinical Research of Endocrinology and Metabolic Diseases in Chongqing, Chongqing University Three Gorges Hospital, Chongqing 404100, China

[3]Department of Endocrinology, Chongqing University Three Gorges Hospital, Chongqing 404100, China

[4]Chongqing Municipality Clinical Research Center for Geriatric diseases, Chongqing University Three Gorges Hospital, Chongqing 404000, China

[#]These authors contributed equally to this project.

[*]**Correspondence:** Xiaohe Luo, Department of Laboratory Medicine, Chongqing University Three Gorges Hospital, No.165, Xincheng Avenue, Wanzhou District, Chongqing 404000, China, Tel +86-13629796135, Email: xiaoheluo@163.com

Supplementary Table S1. Primer sequences for RT-qPCR.

Gene	Species	Forward primer (5'-3')	Reverse primer (5'-3')
TNFRSF11b	Human	TGGTCTCCTGCTAACTCA	CCTGAAGAATGCCTCCTC
Tnfrsf11b	Mouse	GAATGCCGAGAGTGTAGAG	AGAGGTCAATGTCTTGGATG
Cd36	Mouse	AAGAGGTCCTTACACATACAG	CTACAGCCAGATTCAGAACT
Fatp2	Mouse	TTCTCAGCCAGCCAGTT	CATCTCCTCGTAAGCCATT
Fatp4	Mouse	TCAAGTTCCAGAAGACAGAG	TTCCGAGCATCCAGATAGA
Fatp5	Mouse	CTTATTCCTATTGCCTCTACAG	AGTGCTTGCCGCTCTA
Pparγ	Mouse	GGAATTAGATGACAGTGACTTG	AGCACCTTGGCGAACA
Hsl	Mouse	GGCGGCTGTCTAATGTC	TTGGCTGGTGTCTCTGT
Pparα	Mouse	ATGGAGACCTTGTGTATGG	TGGCAGCAGTGGAAGA
Cpt1	Mouse	CAAGCCAGACGAAGAACA	TGACCATAGCCATCCAGAT
Acox1	Mouse	AAGGAAGTGGCGTGGAA	CGGCTCTGTCTTGAATCTT
Mttp	Mouse	GCAGCGTCCACATACAG	GTTCTCCTCTCCTTCATCAG
Srebp1c	Mouse	GCCTGACAGGTGAAATCG	GTCTTGGTTGTTGATGAGC
Acc1	Mouse	AGCAGTTACACCACATACAT	TACCTCAATCTCAGCATAGC
Fasn	Mouse	GTGTGGAAGTTCGTCAGAT	TGTGCTCAGGTTCAGTTG
Scd-1	Mouse	CGTCTGGAGGAACATCATT	CCGAAGAGGCAGGTGTA
Lxrα	Mouse	GGAGCACGCTATGTCTG	TGGCAGGACTTGAGGAG
Lxrβ	Mouse	AACCACGAGACAGAATGC	CGGCTGAGAAGATGTTGAT
Fxr	Mouse	CCAGACAGACAATACATCAAG	TTCACTCTCCAAGACATCAG
Pxr	Mouse	ACCATCGTTCCTGATTCTT	GACCTCCATCTTCCTCCT
Rxrα	Mouse	TCCTTCACCAAGCACATC	ACTCCACCTCGTTCTCAT
Rxrγ	Mouse	GAGAGTGAGGCAGAATGTG	GGAGGCAATGAGCAGTTC
Tnfα	Mouse	CAGGCGGTGCCTATGTCTC	CGATCACCCCGAAGTTCAGTAG
Il6	Mouse	CTGCAAGAGACTTCCATCCAG	AGTGGTATAGACAGGTCTGTTGG
Il1β	Mouse	TTCAGGCAGGCAGTATCACTC	GAAGGTCCACGGGAAAGACAC
Mcp1	Mouse	CTCTTCCTCCACCACCAT	CTCTCCAGCCTACTCATTG
Col1a1	Mouse	CTGGCGGTTCAGGTCCAAT	TTCCAGGCAATCCACGAGC
Col3a1	Mouse	CACAAGGATTACAAGGCATAC	AGGAGCACCGACTTCAC
Ccn2	Mouse	GCACAGAACCACCACTC	GGCACAGGTCTTGATGAA
Tgfb1	Mouse	CCACCTGCAAGACCATCGAC	CTGGCGAGCCTTAGTTTGGAC
Dusp14	Mouse	GGAGACATAGGAGGCATTG	TTGATGACGCAGGTGATG
Rac2	Mouse	AATGTGATGGTGGACAGTAA	GCGAGAAGCAGATGAGAA

Supplementary figures

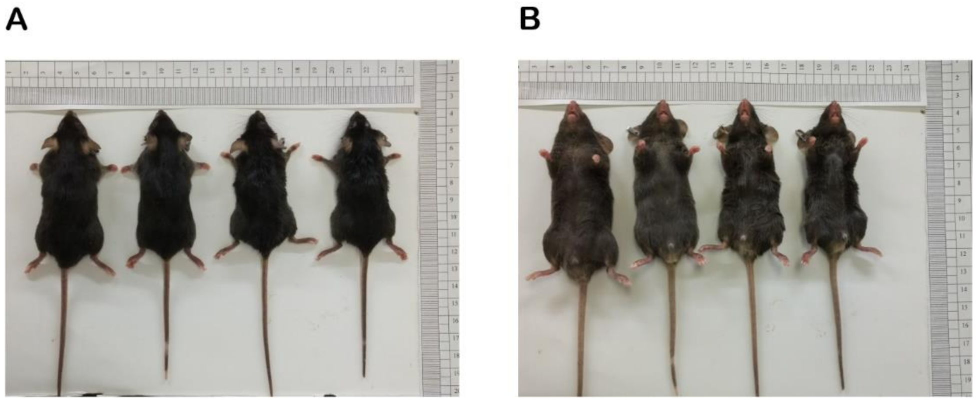

Supplementary Figure S1. Mice macrophotographs. A. Dorsal photograph B. Abdominal photograph

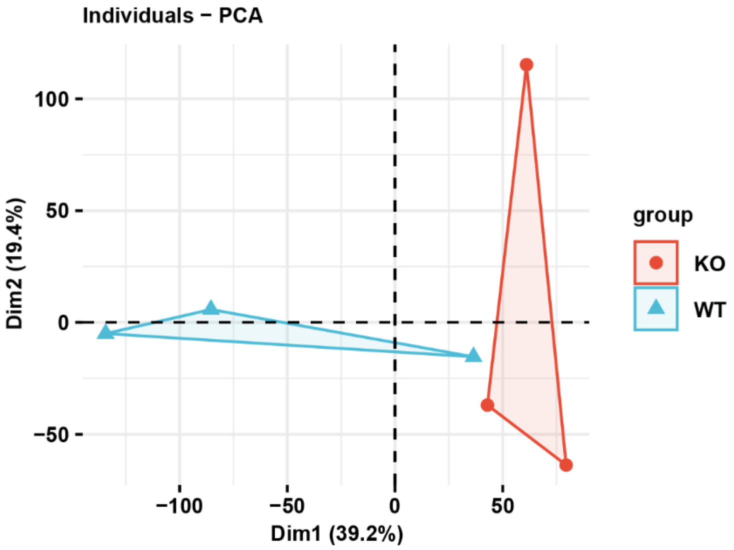

Supplementary Figure S2. Principal component analysis of transcriptome samples.

Supplementary Figure S3. The original uncropped WB bands of Fig 1B.

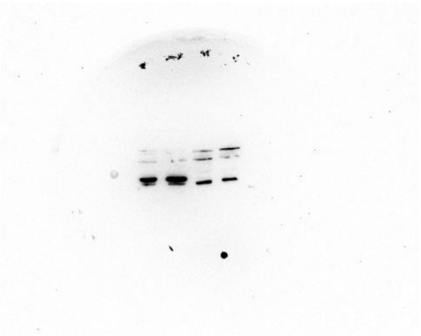

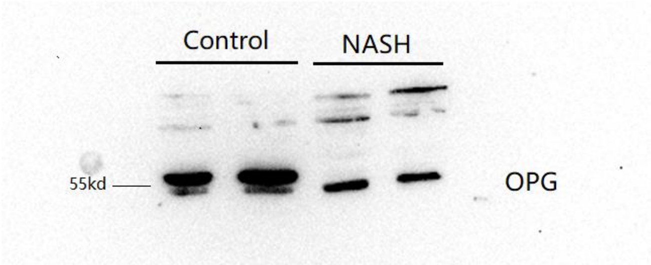

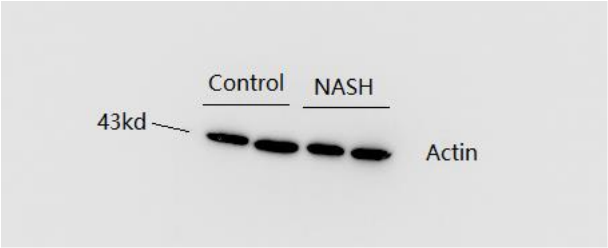

Supplementary Figure S4. The original uncropped WB bands of Fig 1E.

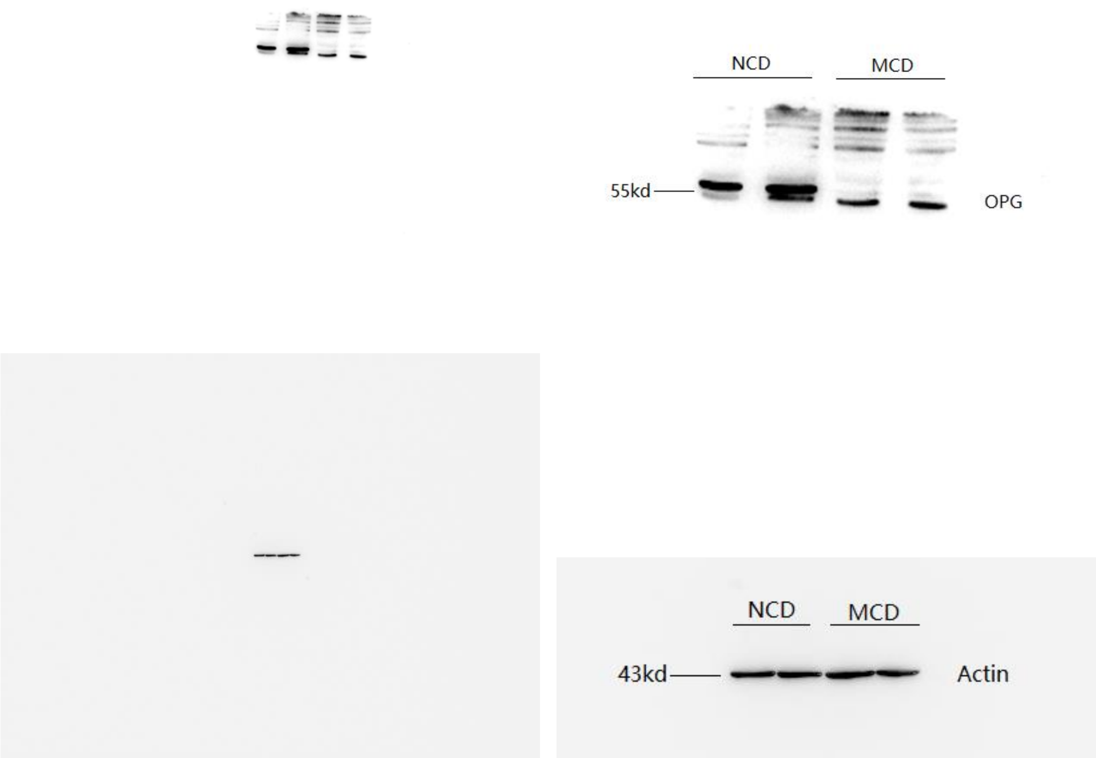

Supplementary Figure S5. **The original uncropped WB bands of Fig 1G.**

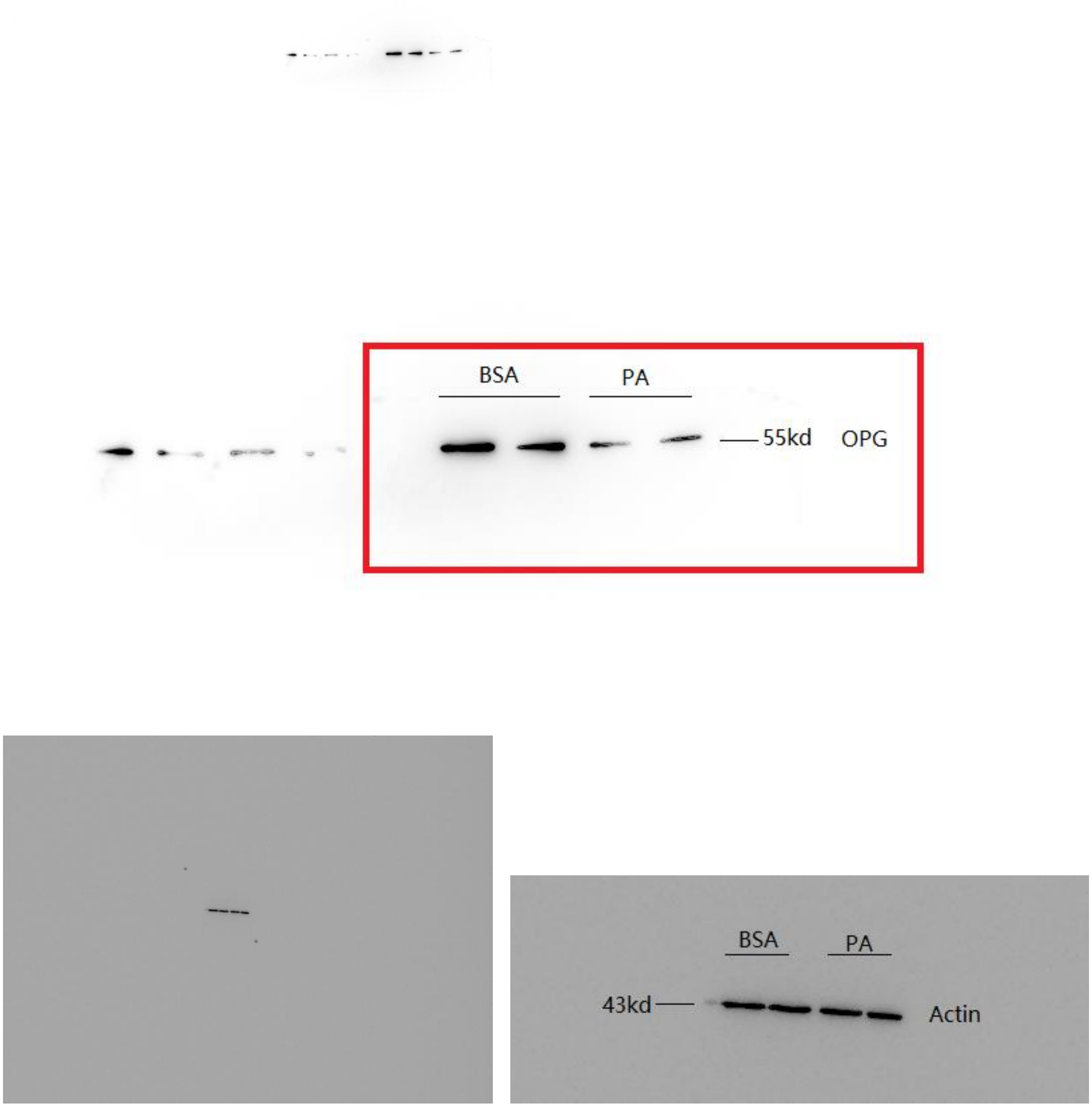

Supplementary Figure S6. **The original uncropped WB bands of Fig 6A.**

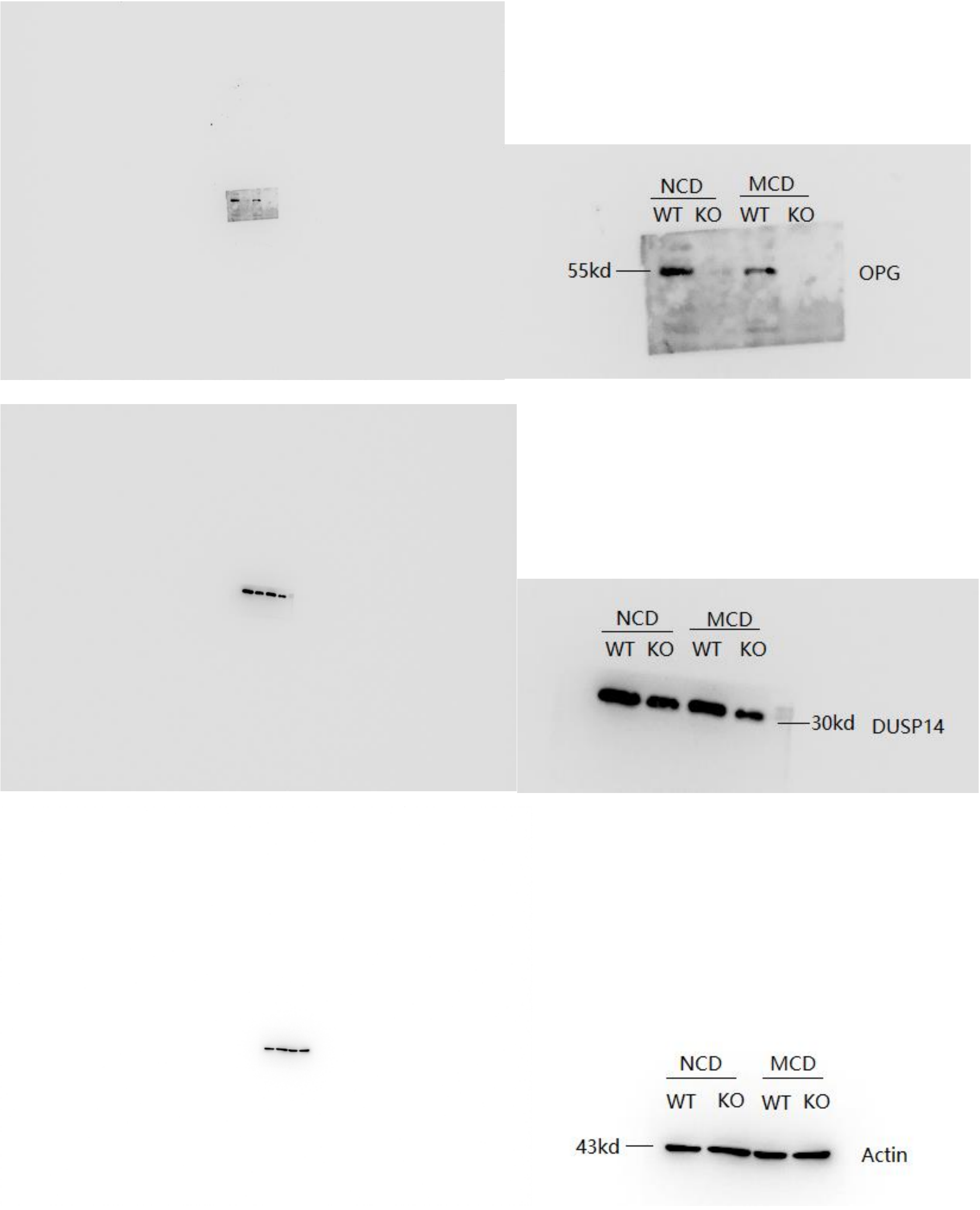

Supplementary Figure S7. **The original uncropped WB bands of Fig 6B.**

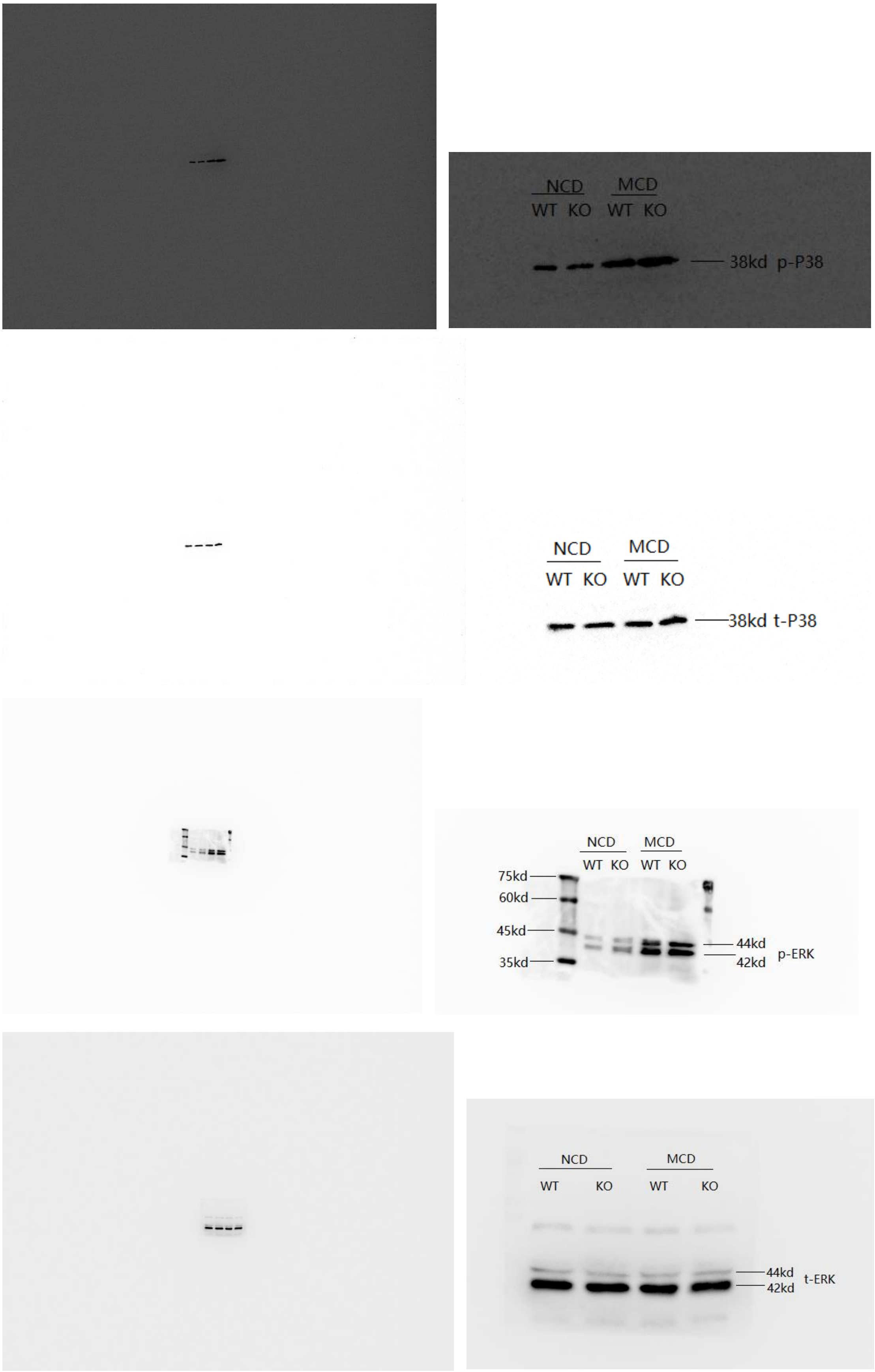

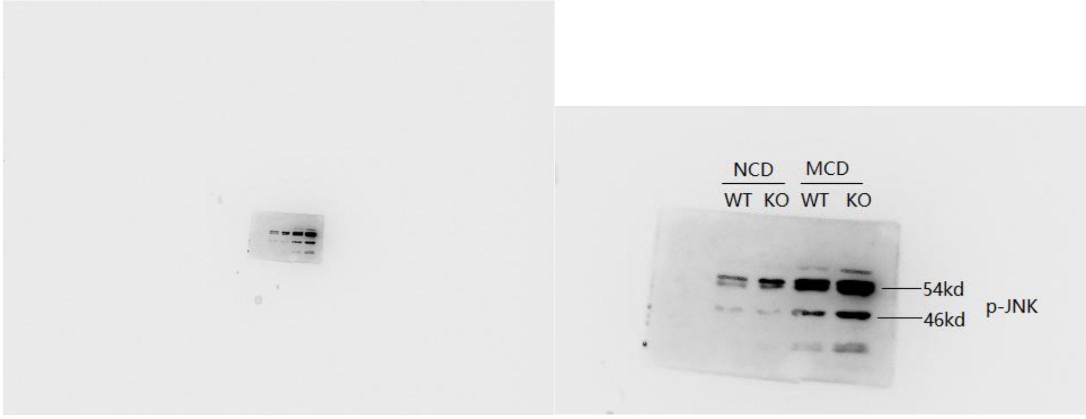
NCD
MCD
WT
KO
WT
KO
54kd
46kd
p-JNK

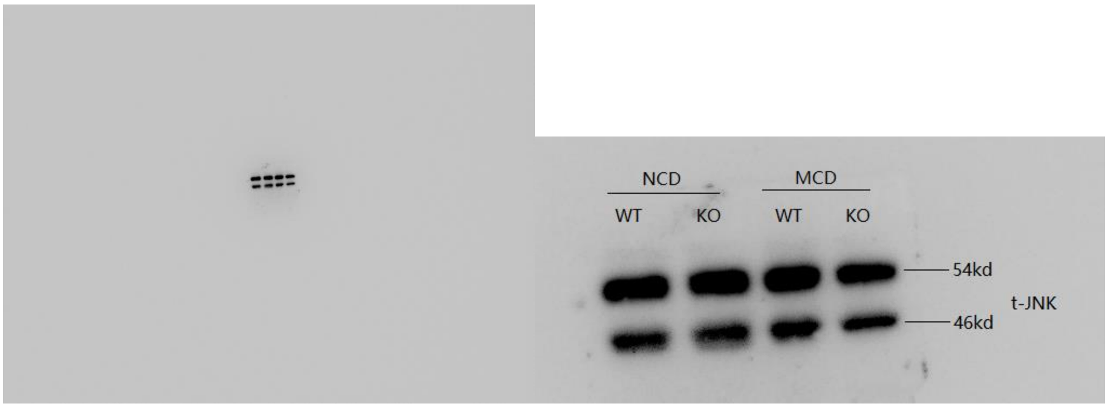
NCD
MCD
WT
KO
WT
KO
54kd
46kd
t-JNK

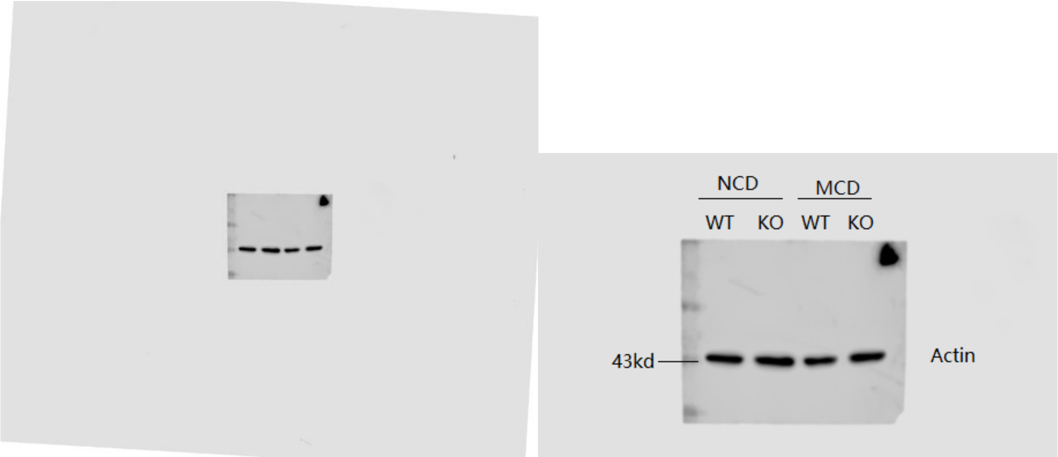
NCD
MCD
WT
KO
WT
KO
43kd
Actin

Supplementary Figure S8. **The original uncropped WB bands of Fig 6C.**

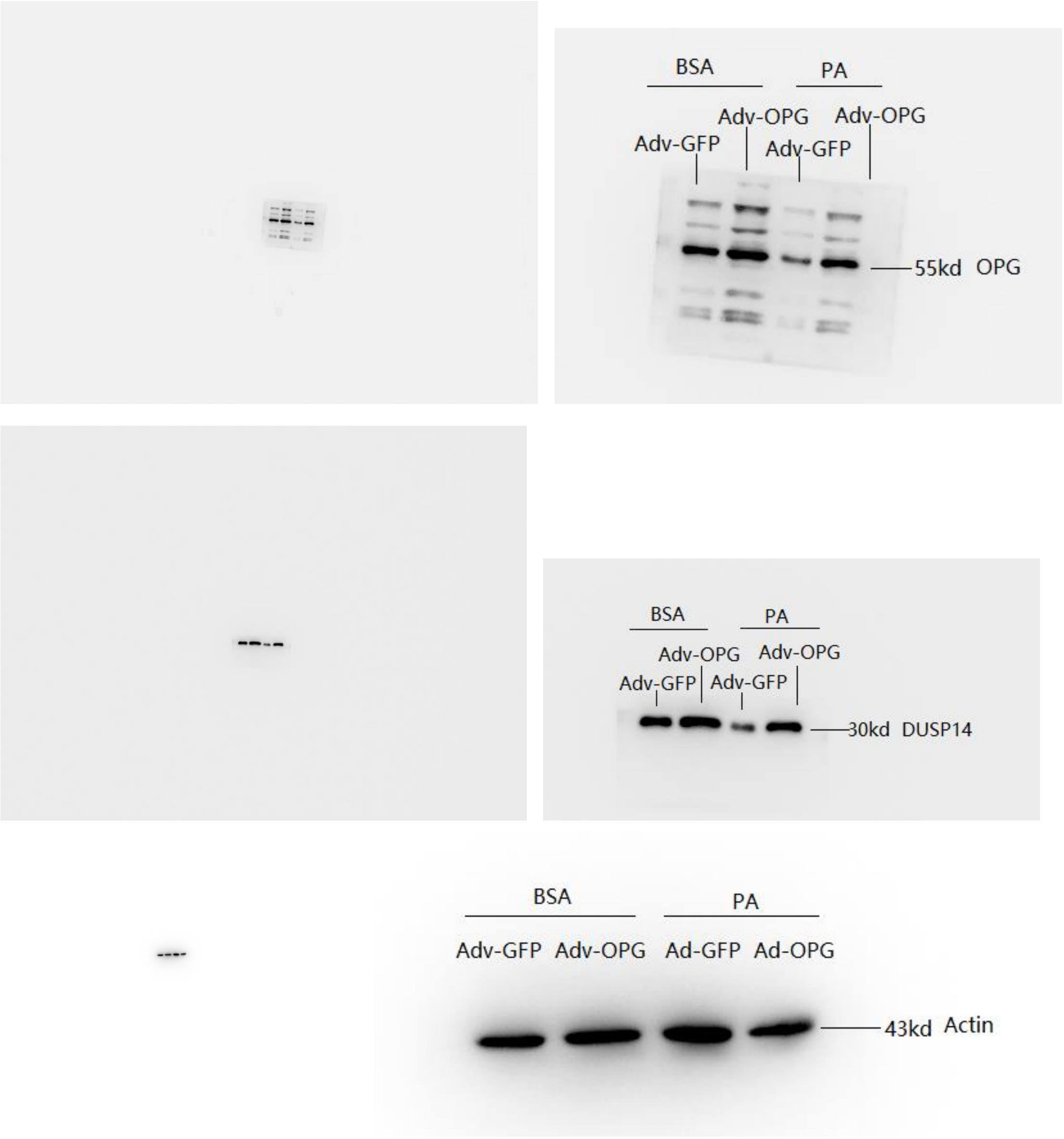

Supplementary Figure S9. **The original uncropped WB bands of Fig 6D.**

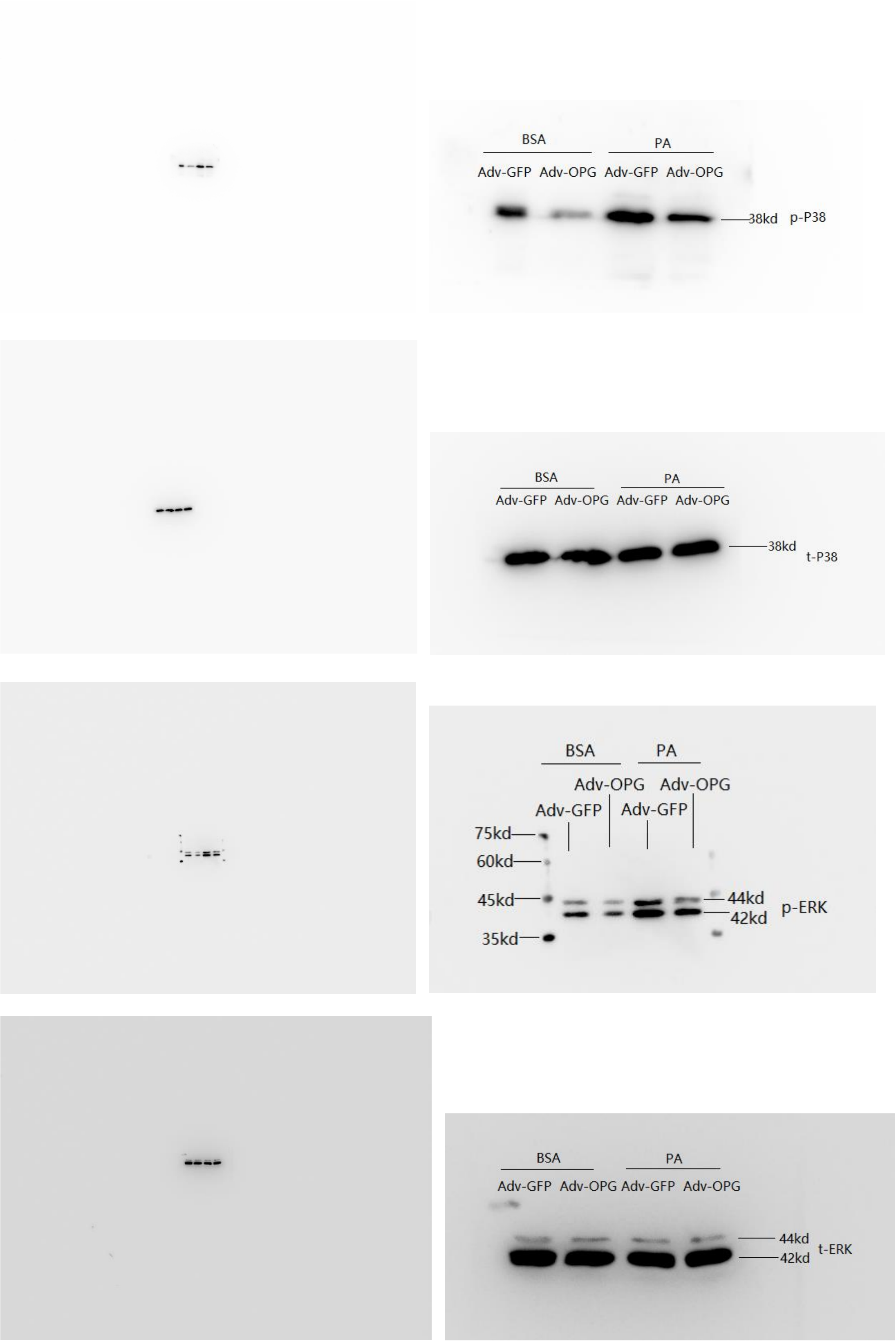

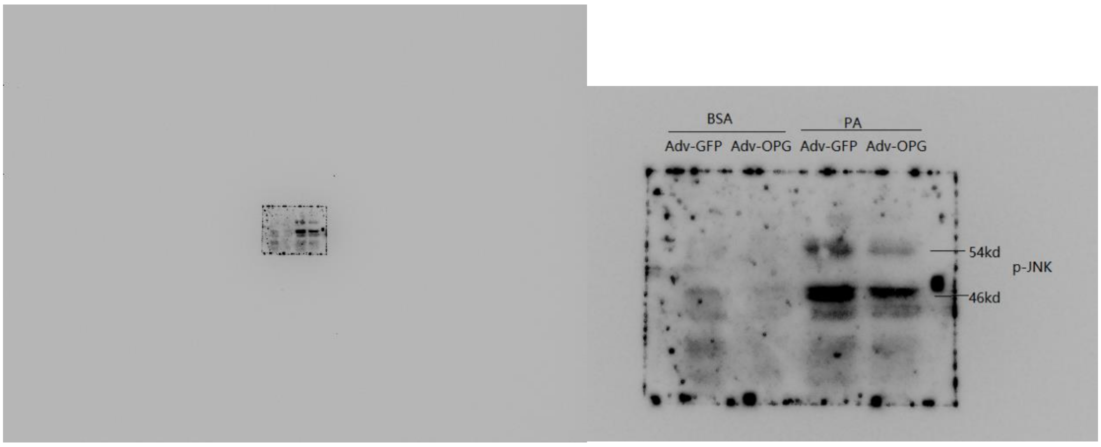
BSA
PA
Adv-GFP Adv-OPG Adv-GFP Adv-OPG
54kd
46kd
p-JNK

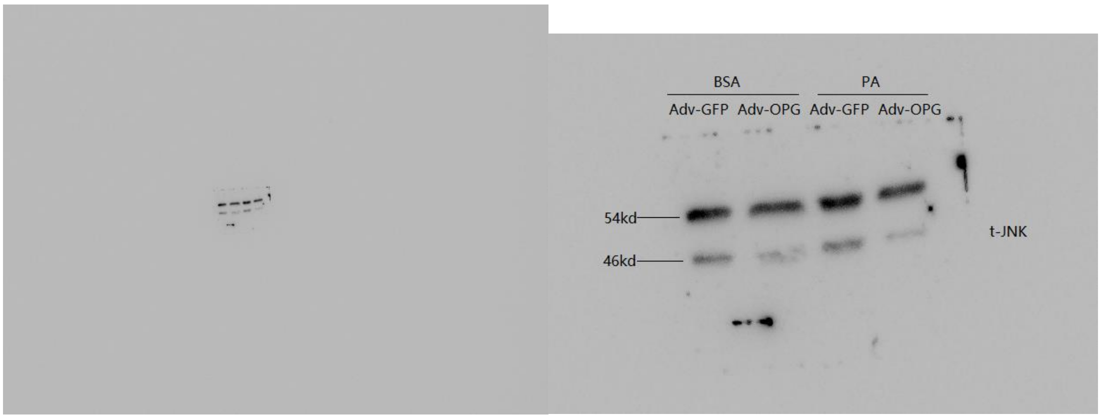
BSA
PA
Adv-GFP Adv-OPG Adv-GFP Adv-OPG
54kd
46kd
t-JNK

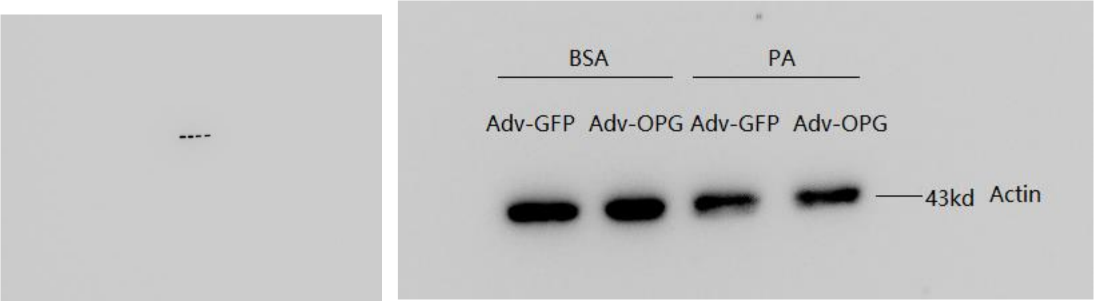
BSA
PA
Adv-GFP Adv-OPG Adv-GFP Adv-OPG
43kd
Actin

Supplementary Figure S10. **The original uncropped WB bands of Fig 6E.**

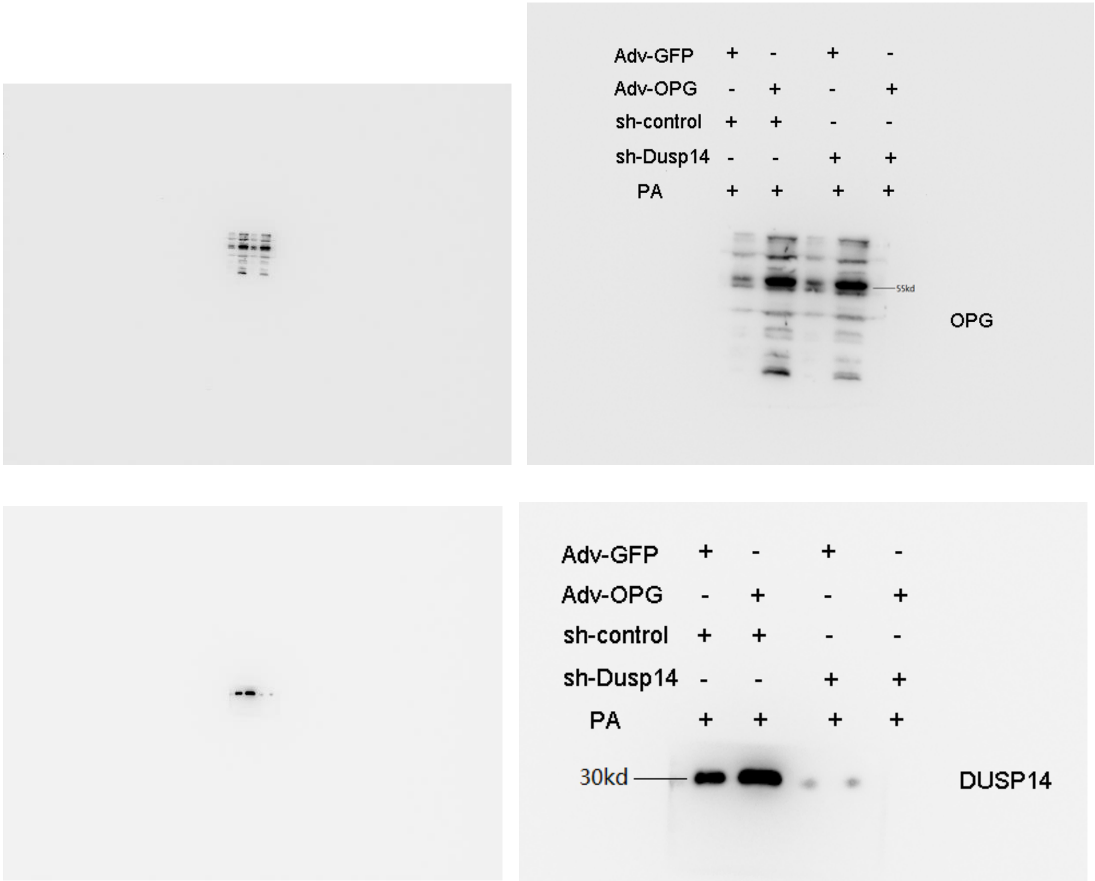

Supplementary Figure S11. **The original uncropped WB bands of Fig 6F.**

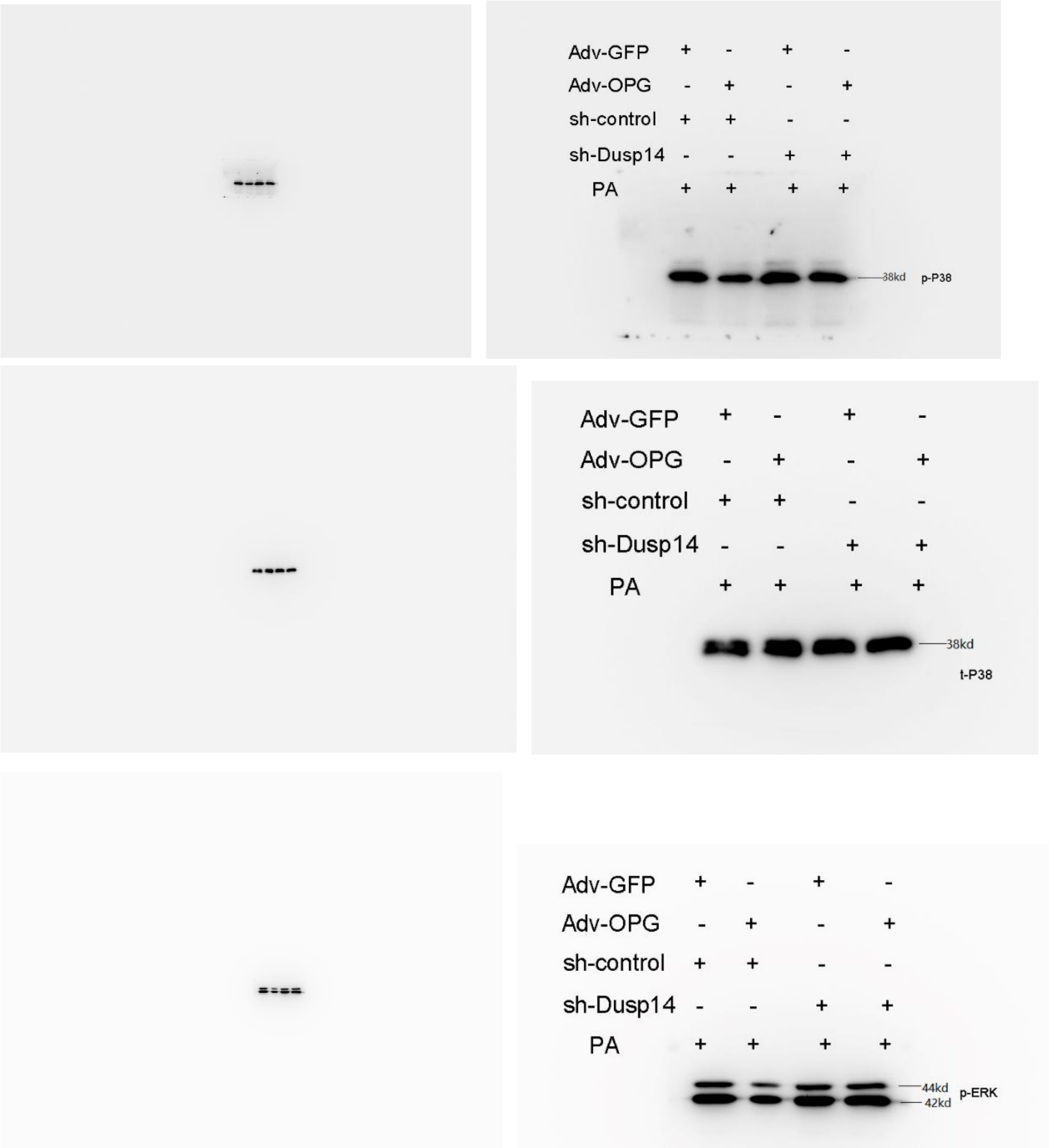

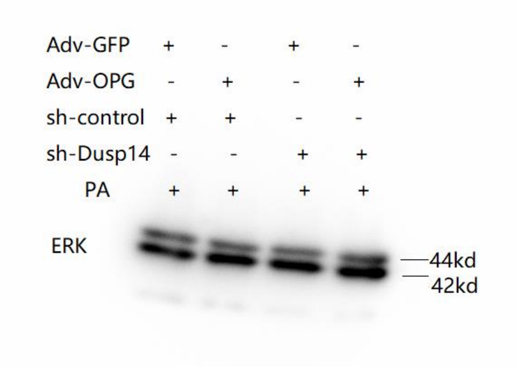

Adv-GFP + - + -
Adv-OPG - + - +
sh-control + + - -
sh-Dusp14 - - + +
PA + + + +
ERK
44kd
42kd

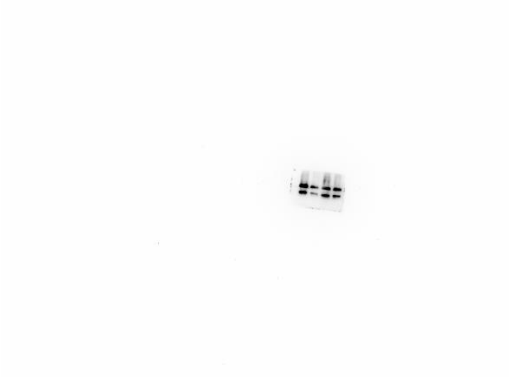

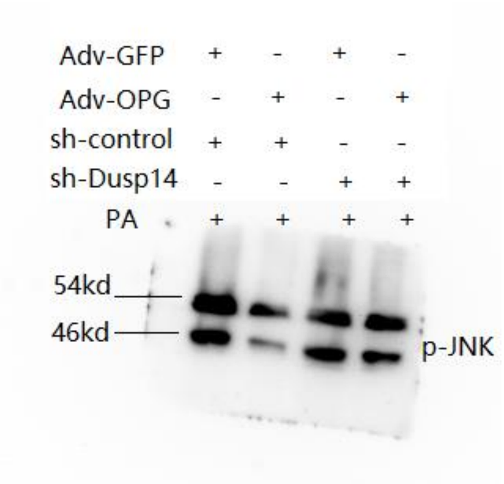

Adv-GFP + - + -
Adv-OPG - + - +
sh-control + + - -
sh-Dusp14 - - + +
PA + + + +
54kd
46kd
p-JNK

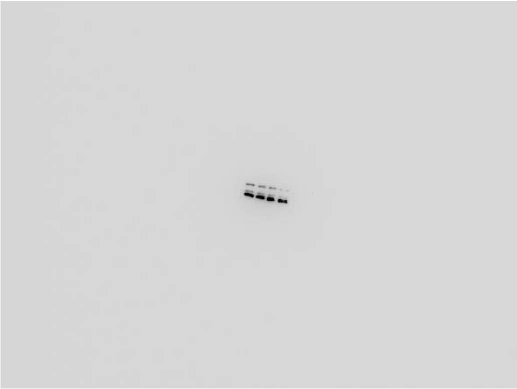

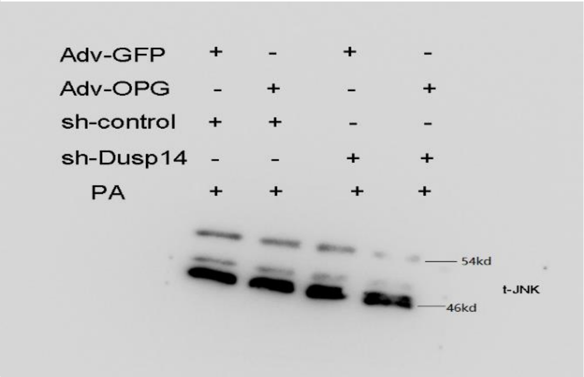

Adv-GFP + - + -
Adv-OPG - + - +
sh-control + + - -
sh-Dusp14 - - + +
PA + + + +
54kd
t-JNK
46kd

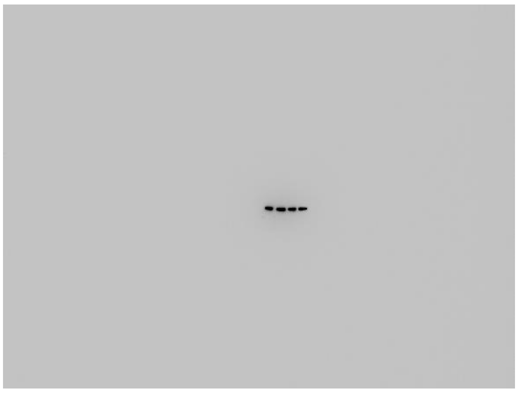

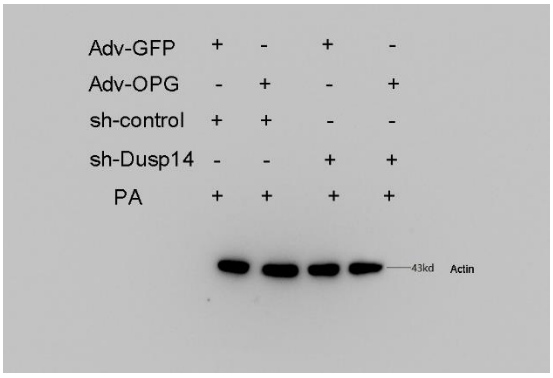

Adv-GFP + - + -
Adv-OPG - + - +
sh-control + + - -
sh-Dusp14 - - + +
PA + + + +
43kd
Actin
